# Phthalate mixtures in pregnancy, autistic traits, and adverse childhood behavioral outcomes

**DOI:** 10.1016/j.envint.2020.106330

**Published:** 2021-01-05

**Authors:** Drew B. Day, Brent R. Collett, Emily S. Barrett, Nicole R. Bush, Shanna H. Swan, Ruby H.N. Nguyen, Adam A. Szpiro, Sheela Sathyanarayana

**Affiliations:** a Center for Child Health, Behavior, and Development, Seattle Children’s Research Institute, 1920 Terry Ave, Seattle, WA 98101, USA; b Department of Psychiatry and Behavioral Sciences, University of Washington, 1959 NE Pacific Street, Seattle, WA 98195, USA; c Department of Epidemiology, Environmental and Occupational Health Sciences Institute, Rutgers School of Public Health, 170 Frelinghuysen Road, Piscataway, NJ 08854, USA; d Center for Health and Community, Department of Psychiatry and Behavioral Sciences, Weill Institute for Neurosciences, Department of Pediatrics, University of California, San Francisco, 401 Parnassus Avenue, San Francisco, CA 94143, USA; e Department of Environmental Medicine and Public Health, Icahn School of Medicine at Mount Sinai, 17 E. 102nd Street, CAM Building, 3 West, One Gustave L. Levy Place, New York, NY 10029, USA; f Department of Epidemiology and Community Health, University of Minnesota, 420 Delaware Street Southeast, Minneapolis, MN 55455, USA; g Department of Biostatistics, University of Washington, 1705 Northeast Pacific Street, Seattle, WA 98195, USA; h Department of Pediatrics, University of Washington, 1959 Northeast Pacific Street, Seattle, WA 98195, USA

**Keywords:** Phthalates, Autism, Child behavior, Mixture effects, Prenatal exposure, Externalizing behavior

## Abstract

**Background:**

Prenatal exposure to multiple phthalates is ubiquitous, and yet few studies have evaluated these exposures as a mixture in relation to child autistic traits and behavioral problems.

**Objectives:**

To assess cumulative associations between prenatal phthalate mixtures and child behaviors, including effect modification by exposure timing and child sex.

**Methods:**

Analyses included 501 mother/child pairs from the multicenter pregnancy cohort The Infant Development and Environment Study (TIDES). Nine maternal urinary phthalate metabolites were measured in early and late pregnancy, and behavior was assessed at ages 4–5 years using composite T scores for the Behavioral Assessment System for Children (BASC-2), which measures several dimensions of child behavior, and the Social Responsiveness Scale (SRS-2), which measures social impairment consistent with autistic traits. We utilized weighted quantile sum (WQS) regressions to examine pregnancy period-specific associations between phthalate mixtures and behavioral outcomes. Full-sample 95% WQS confidence intervals are known to be anticonservative, so we calculated a confirmatory p-value using a permutation test. Effect modification by sex was examined with stratified analyses.

**Results:**

A one-quintile increase in the early pregnancy phthalate mixture was associated with increased SRS-2 total score (coefficient = 1.0, confirmatory p = 0.01) and worse adaptive skills (coefficient = −1.0, confirmatory p = 0.06) in both sexes. In sex-stratified analyses, the early pregnancy phthalate mixture was associated with increased SRS-2 total score in boys (coefficient = 1.2, confirmatory p = 0.04) and girls (coefficient = 1.0, confirmatory p = 0.10) and worse BASC-2 adaptive skills score in girls (coefficient = −1.5, confirmatory p = 0.06), while the late pregnancy phthalate mixture was associated with increased BASC-2 externalizing score in boys (coefficient = 1.3, confirmatory p = 0.03).

**Conclusion:**

Our results suggest cumulative adverse associations between prenatal phthalate mixtures and multiple facets of childhood behavior.

## 1. Introduction

Phthalates are non-persistent, ubiquitous industrial chemicals that can cross the placenta and affect fetal neurodevelopment (Silva et al. 2004). They are endocrine-disrupting chemicals that can affect normal sex hormone homeostasis in the body. Sex hormones such as testosterone and estrogen play an important role in sex-specific neurobehavioral development, and phthalates have been shown to inhibit sex hormone signaling in murine brains (Xu et al. 2015). Animal studies have identified additional mechanisms by which phthalates could potentially influence neurodevelopment, including decreased dopaminergic activity in the brain (Tanida et al. 2009), and the disruption of calcium signaling, lipid metabolism, and thyroid hormone levels, the latter of which has been supported by several epidemiologic studies as well (Miodovnik et al. 2014; Tanida et al. 2009). Pregnant women are exposed to a complex mixture of phthalates on a daily basis (Woodruff et al. 2011), with major sources varying by compound and including oral exposure through the diet (Serrano et al. 2014), dermal exposure to personal care products (Buckley et al. 2012), and inhalation of house dust (Adibi et al. 2008). Several animal studies have found cumulative effects of phthalate mixtures on reproductive development, testosterone production, and fetal mortality, suggesting an additive mixture effect (Howdeshell et al. 2008a; Howdeshell et al. 2008b; Rider et al. 2008). Though real-life phthalate exposures occur in mixtures, to date few studies have examined phthalate mixtures in relation to behavioral outcomes.

Studies of prenatal phthalate metabolites and child neurodevelopment generally show adverse associations, though these findings are somewhat mixed in terms of specific associations for each phthalate and difficult to interpret due to variations in the metabolite exposures studied, the timing of assessment, and the outcomes assessed (Ejaredar et al. 2015; Zhang et al. 2019). A recent meta-analysis found only slight or indeterminate evidence of individual gestational phthalate associations with child behavior and other neurocognitive outcomes, noting that the inconsistency across these findings may be due in part to analyzing phthalates individually rather than as a mixture, as well as inadequate assessment of sex-specific effects and potential critical windows of exposure (Radke et al. 2020).

In this study, we address these gaps in the literature by using weighted quantile sum (WQS) regression to examine associations between maternal prenatal phthalates measured in early pregnancy and late pregnancy and child neurobehavioral outcomes at ages 4–5 years. WQS regression estimates the cumulative association of the exposure mixture with outcomes, as well as the weighted contribution of each individual exposure in the mixture to the combined association (Czarnota et al. 2015), a significant advance from analyzing one chemical at a time. Our behavioral outcomes included parent-reported externalizing, internalizing, adaptive behavior, and autistic traits. We also examined sex-specific effects in stratified analyses. We hypothesized that prenatal maternal phthalate mixtures would be associated with child behavior problems, with associations differing for mixture exposures in the early and late pregnancy periods.

## 2. Methods

### 2.1. Study participants

The Infant Development and Environment Study (TIDES) is a prospective cohort of pregnant women recruited in their first trimester from 2010 to 2012 at the University of California, San Francisco (UCSF); University of Minnesota (UMN); University of Rochester Medical Center (URMC); and Seattle Children’s Hospital/University of Washington (UW). Study design and methods have been previously published (Barrett et al. 2014). Informed consent was obtained from all recruited participants, and questionnaires including items on demographics, medical history, psychosocial stressors, and lifestyle were administered in each of the three trimesters. Out of 801 mothers who completed at least one assessment during pregnancy, this analysis is limited to 500 mothers and 501 children, including one pair of twins, with complete data for all 9 phthalates in early and/or late pregnancy, child 4–5-year behavioral assessments, and covariate data.

### 2.2. Urinary phthalate measurements

Spot urine samples were collected at two time points at study center clinics: in early pregnancy between gestational weeks 6 and 21 (mean = 11 weeks gestation) and late pregnancy between gestational weeks 26 and 42 (mean = 33 weeks gestation) using previously described methods (Ferguson et al. 2019; Swan et al. 2015). Measurements were defined by these broad time periods rather than strictly as trimesters, as neurodevelopmental windows of susceptibility may not be captured by trimesters (Howdeshell 2002). Immediately following urine collection, dilution was assessed by measuring specific gravity with a handheld refractometer, after which samples were stored at −80 ^°^C until analysis. Samples were analyzed over time in subsets, the first being the early pregnancy samples, which were initially analyzed by the Division of Laboratory Sciences, National Center for Environmental Health, Centers for Disease Control and Prevention (CDC) and the University of Washington (UW) as previously described using a method involving enzymatic deconjugation of phthalates from glucuronidated forms, solid phase extraction, separation with high performance liquid chromatography (HPLC), and detection by either isotope-dilution tandem mass spectrometry (CDC) or electrospray ionization-tandem mass spectrometry (Silva et al. 2007; Swan et al. 2015). For late pregnancy, samples were analyzed by the CDC using the aforementioned methods. Phthalate concentrations below the limit of detection (LOD) were imputed using the LOD divided by $\sqrt{2}$ (Hornung and Reed 1990). Table A.1 in the Supplemental Material lists the measured phthalate metabolites and their respective parent compounds.

### 2.3. Neurobehavioral assessments

The Behavioral Assessment System for Children, 2nd Edition (BASC-2) and the Social Responsiveness Scale, 2nd Edition (SRS-2) were completed by mothers of TIDES children between 4 and less than 6 years of age. The BASC-2 provides four composite scores: externalizing, internalizing, behavioral symptoms index, and adaptive skills composite scores. The BASC-2 externalizing composite score is comprised of hyperactivity and aggression subscale scores; the internalizing composite is comprised of anxiety, depression, and somatization subscale scores; the behavioral symptoms index (BSI) is comprised of attention problems, atypicality, and withdrawal subscale scores; and the adaptive skills composite is comprised of activities of daily living, adaptability, functional communication, and social skills subscale scores. Higher scores indicate more problematic behaviors for all BASC-2 scores except for the adaptive skills scores. The SRS-2 provides a total composite score indicating social communication deficits and restrictive behaviors that can be used to screen for autism (Frazier et al. 2012) or to capture broader variation in social impairment across unaffected samples (Constantino and Gruber 2012). Higher SRS-2 scores indicate more problematic behaviors. For both BASC-2 and SRS-2, raw scores were converted into sexand age-standardized, norm-referenced T scores (mean = 50, SD = 10) based on each assessment’s manualized procedures. As parent-reported measures can be influenced by parental emotional state (Fergusson et al. 1993), maternal depression and stress were also assessed in the same visit using the Patient Health Questionnaire (PHQ-9) (Wittkampf et al. 2007) and Perceived Stress Scale (PSS-10) (Cohen et al. 1983), respectively.

### 2.4. Statistical methods

Associations between maternal urinary phthalate mixtures and child behavioral outcomes were analyzed separately between early and late pregnancy. Phthalate measurements with concentrations above the LOD in at least 50% of the samples or for which no more than 20% of the samples were missing for either pregnancy period were included in this analysis and listed in Table 2. We used BASC-2 and SRS-2 composite scores as outcomes, analyzed as sex-standardized T scores. Maternal urinary phthalate concentrations were adjusted for specific gravity using the Levine-Fahy equation (Levine and Fahy 1945), and they were log_10_-transformed in all models due to right skew. We chose to include the highly correlated DEHP metabolites MEHP, MEOHP, MEHHP, and MECPP separately in mixture models rather than as one sum measure of DEHP metabolites to avoid the potential loss of information on differential toxicity of individual metabolites.

Weighted quantile sum (WQS) regression was used to examine associations. Log_10_-transformed, specific gravity-adjusted phthalates were transformed into quintiles because quintiles provided a balance between having quantiles large enough to include all < LOD values for most metabolites but not being so large as to lose resolution. Median levels and variability differed for certain metabolites across study centers, and so to address this heterogeneity we used study center-specific quintiles. WQS regressions iteratively select weights that optimize the likelihood of the model when the coefficient for the sum mixture is positive or negative, and so we ran each model twice, once for each direction, in order to characterize potential positive and negative mixture associations. To ensure estimate stability, we excluded results for a particular direction if fewer than 100 bootstrapped weights out of 1000 total bootstraps were associated with sum mixture coefficients in that direction. The full-sample confidence intervals from the WQS regression were calculated using the “HC0” Huber-White heteroskedasticity-consistent standard error sandwich estimator (White 1980). To increase statistical power, we did not split the data into training and validation datasets for weight and coefficient estimation, though this can also give confidence intervals that are anticonservative estimates of the true precision. Therefore, we applied a permutation test (Freedman and Lane 1983) to obtain p-values that more accurately estimate uncertainty in the WQS coefficient (Loftus et al. 2020). To examine potential differences in mixture coefficients and weights by sex, WQS regressions stratified by child sex were also evaluated.

All models adjusted for the a priori-selected covariates maternal and child age, pre-pregnancy BMI, study center, race, income, maternal education, child sex, parity (primiparous vs. multiparous), time of the day and gestational age at urine collection, self-reported alcohol and cigarette consumption during pregnancy, and maternal depression and stress as measured by mean PHQ-9 and PSS-10 score, respectively. Covariates were chosen a priori because of their potential confounding effect on one or more outcome measures (Fox et al. 1995; Herrmann et al. 2008; O’Connor and Paley 2009) or for potential impacts on exposure levels (Arbuckle et al. 2016). We controlled for the timingdependent covariates time of the day and gestational age at urine collection to adjust for potential exposure patterns varying by day or within pregnancy periods (Li et al. 2019). Based on analysis of timingdependent covariate associations with phthalates, we chose to include gestational age at urine collection as a linear variable and time of the day of urine collection as a natural cubic spline with 3 degrees of freedom. We also used inverse probability weighting (IPW) (Robins et al. 1994) in sensitivity analyses to examine the effects of attrition bias (see Results A.4). Individual phthalate regression results are also presented for reference (Results A.7).

## 3. Results

### 3.1. Study participants

501 participants had complete data for phthalates in at least one pregnancy period, at least one behavioral outcome, and all covariates. Demographic characteristics for these participants included in the final analysis are shown in Table 1, and characteristics are compared between included and excluded participants in Table A.3. Mothers included in the final analysis were more likely to be older, white, and have a higher income and education level. Maternal PSS-10 scores were typical of values observed in women of a similar age in the United States (Cohen and Janicki-Deverts, 2012). PHQ-9 scores corresponded with clinical cutoffs that primarily indicated minimal (n = 389 (77.6%)) or mild depression (83 (16.6%)), with only small numbers of participants exhibiting moderate (19 (3.8%)), moderate-severe (6 (1.2%)), and severe depression (4 (0.8%)). Urine collection times of the day did not significantly differ between pregnancy periods (paired *t*-test p = 0.2).

### 3.2. Phthalate metabolite concentrations

Individual phthalate metabolite concentrations (Table 2) were typical for pregnant women across the United States (Daniel et al. 2020; Woodruff et al. 2011). Compared with the early pregnancy period, late pregnancy concentrations were significantly lower for MEHP and MEHHP; as well as significantly higher for MEP, MBP, MiBP, and MECPP; with percent differences ranging from 13 to 40%. Boxplots comparing metabolite concentrations and quintile break points across study centers, sexes, and pregnancy periods are shown in Fig. A.1, and LOD values are shown in Table A.2. Log_10_-transformed concentrations were moderately to highly correlated within each pregnancy period (see Results A.3), with the highest correlations being between the DEHP metabolites (r = 0.62 – 0.98). Correlations between early and late pregnancy phthalate metabolite concentrations were lower, with lower molecular weight metabolites being moderately correlated (r = 0.34 – 0.46) and DEHP metabolites and MCPP being largely uncorrelated (r = 0.08 – 0.19).

### 3.3. Behavior outcomes

BASC-2 externalizing and BSI scores were highly correlated, r = 0.86 (see Fig. A.6). Therefore, we focused on externalizing score in our main results (BSI results are presented in Results A.5). Though BASC-2 and SRS-2 T scores are sex-standardized at the population level, males had significantly lower BASC-2 internalizing and higher SRS-2 total composite T scores (Table 3; see boxplots in Fig. A.2).

### 3.4. WQS regressions analyzing the total population

**Fig. 1.**
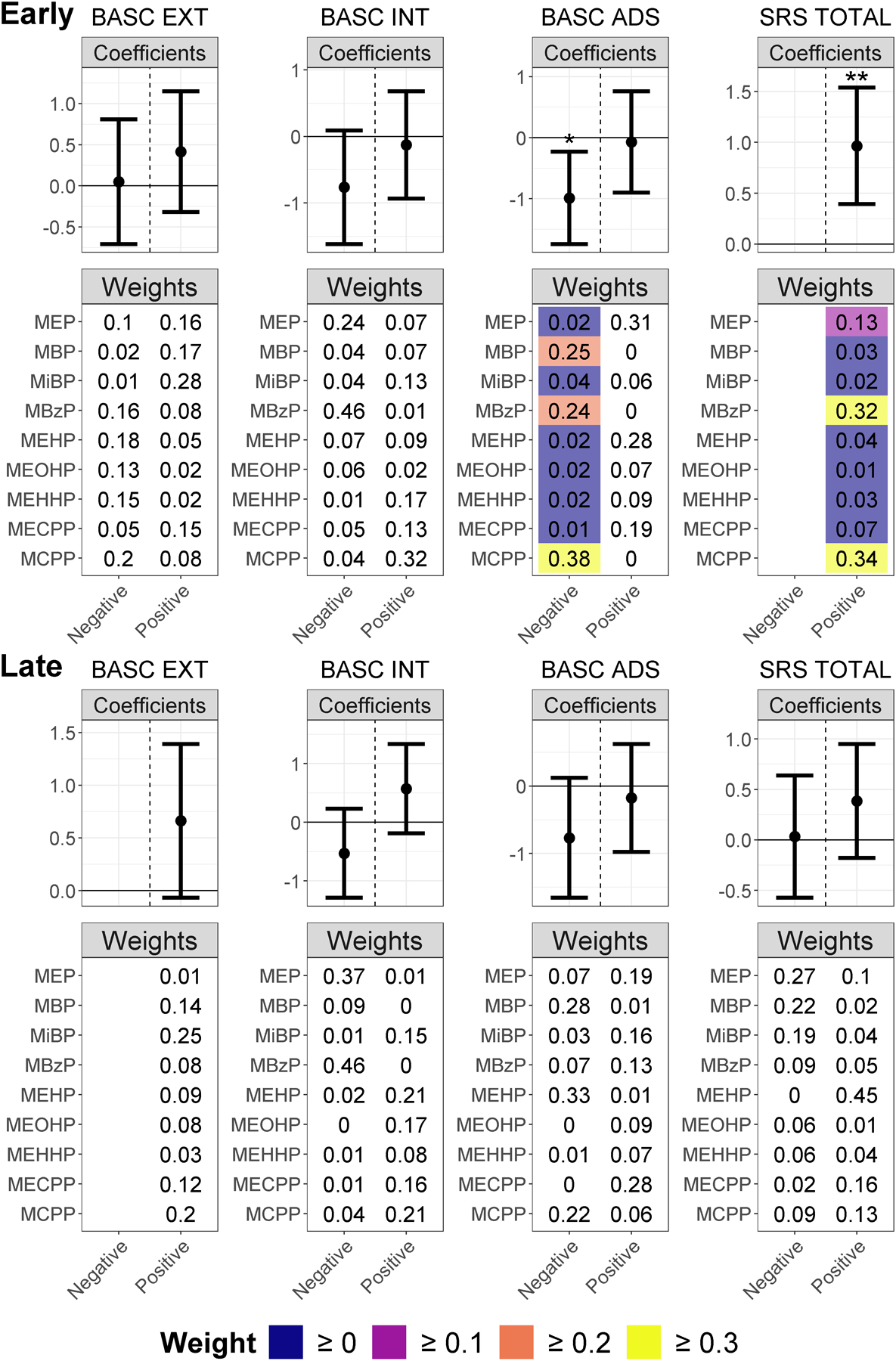
WQS Regression Coefficients and Weights for Associations between Phthalate Mixtures in Early and Late Pregnancy and Behavior in the Total Population. Weighted quantile sum coefficient means and 95% CIs for the negative and positive directions are presented in the forest plots (labelled “Coefficients”). Coefficients significant in the WQS regressions are denoted with “*”, and coefficient significant after the permutation test are denoted with “**”. The heatmap plots labelled “Weights” show the weighted quantile sum weights for all phthalates in the mixture, with weights only color-coded if the full-sample 95% CIs for the WQS coefficient do not overlap zero and higher weights display a lighter color. We de-emphasized the weights for WQS coefficients with full-sample 95% CIs overlapping zero by not color-coding them because when the WQS coefficient is zero or not statistically significantly different from zero, the weights do not convey useful information, as they show partial contribution to a sum coefficient of zero. WQS coefficients and weights based on fewer than 100 usable bootstraps are omitted from the figure since they are unstable estimates.

WQS regression coefficients show the magnitude of the association either in the positive or negative direction between the mixture and outcomes, and higher weights reflect a greater contribution of those phthalates to the cumulative association. All coefficient results are listed in Table A.5. Fig. 1 shows that in early pregnancy, a one quintile increase in the weighted quantile sum of the phthalate mixture was associated with a 1.0-point increase in SRS-2 total score with full-sample CIs not overlapping zero (full-sample 95% CI: 0.4, 1.5), and the highest weights were for MCPP, MBzP, and MEP. The confirmatory permutation test p-value confirmed this association as significant (confirmatory p = 0.010). We observed an additional association between a phthalate mixture heavily weighted for MCPP, MBP, and MBzP in early pregnancy and a 1.0-point (full-sample 95% CI: 0.2, 1.7) decrease in BASC-2 adaptive skills score that was nonsignificant but suggestive when applying the permutation test (confirmatory p = 0.055). Additionally, there was a suggestive mixture association with increased BASC-2 internalizing score.

In late pregnancy, all observed associations had full-sample CIs that overlapped zero, though there were suggestive mixture associations with worsened BASC-2 externalizing behavior and adaptive skills scores. IPW results were similar (see Fig. A.7).

### 3.5. WQS regressions stratified by child sex

**Fig. 2.**
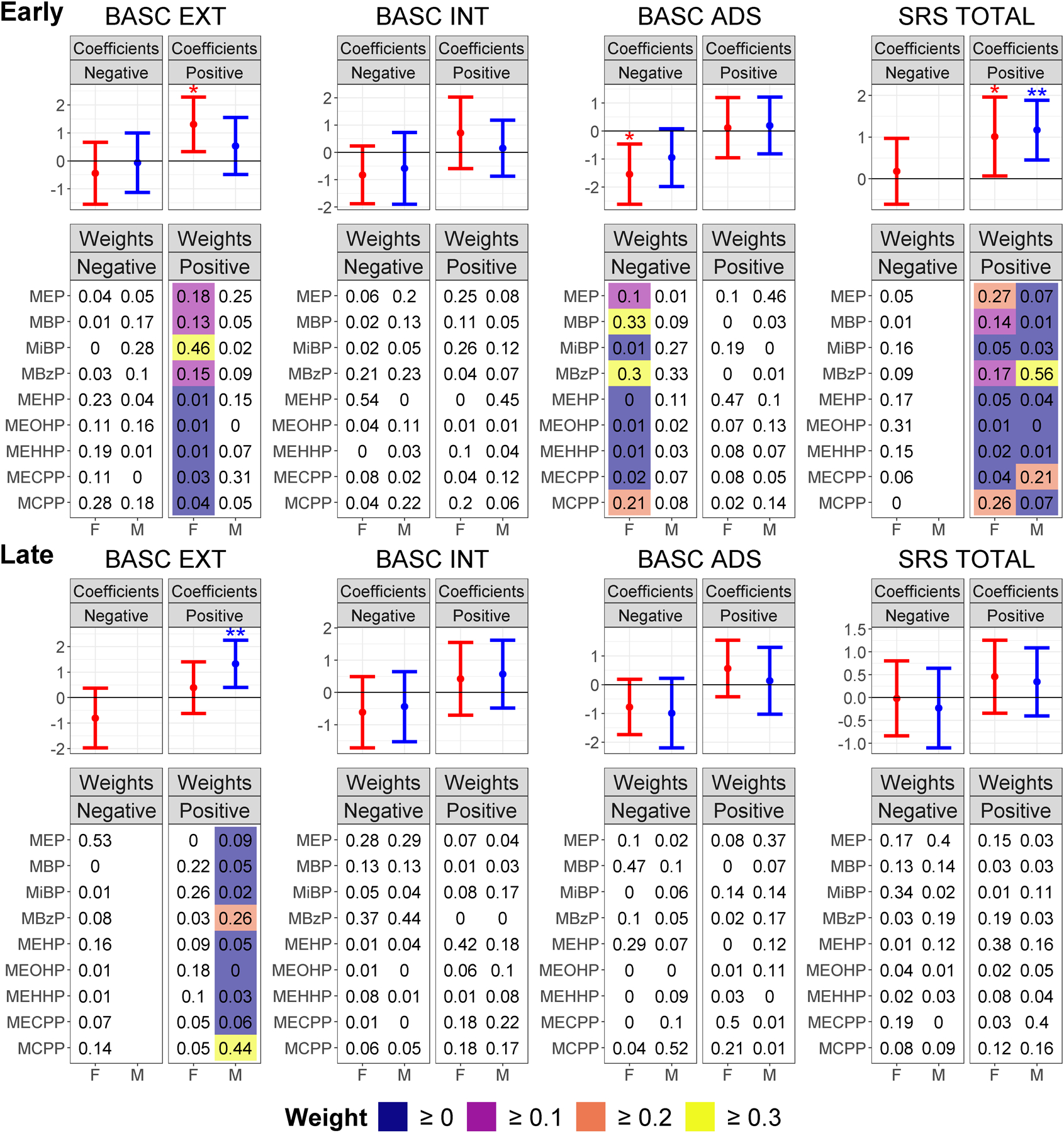
WQS Regression Coefficients and Weights for Associations between Phthalate Mixtures in Early and Late Pregnancy and Behavior Stratified by Sex. Weighted quantile sum coefficient means and 95% CIs for the negative and positive directions are presented in the forest plots (labelled “Coefficients”). Coefficients significant in the WQS regressions are denoted with “*”, and coefficients significant after the permutation test are denoted with “**”. Male and female coefficients are colored blue and red, respectively. The heatmap plots labelled “Weights” show the weighted quantile sum weights for all phthalates in the mixture, with weights only color-coded if the full-sample 95% CIs for the WQS coefficient do not overlap zero and higher weights display a lighter color. We de-emphasized the weights for WQS coefficients with full-sample 95% CIs overlapping zero by not color-coding them because when the WQS coefficient is zero or not statistically significantly different from zero, the weights do not convey useful information, as they show partial contribution to a sum coefficient of zero. WQS coefficients and weights based on fewer than 100 usable bootstraps are omitted from the figure since they are unstable estimates.

Fig. 2 shows that in early pregnancy, associations between SRS-2 total score and early pregnancy phthalate mixtures were similar in magnitude in boys and girls (coefficients = 1.2 (full-sample 95% CI: 0.4, 1.9) and 1.0 (full-sample 95% CI: 0.1, 2.0), respectively). However, the mixture weights for boys were higher for MBzP and MECPP; and those for girls were higher for MEP, MCPP, MBzP, and MBP. Also, the male-specific association with SRS-2 total score was significant using the permutation test (confirmatory p = 0.025), but that was not true for the association in girls (confirmatory p = 0.105). Specific to girls, there was also a 1.5-point (full-sample 95% CI: 0.5, 2.6; confirmatory p = 0.055) decrease in BASC-2 adaptive skills score and a 1.3-point (full-sample 95% CI: 0.3, 2.3; confirmatory p = 0.171) increase in BASC-2 externalizing score associated with the phthalate mixture.

In late pregnancy and specific to boys, there was a significant 1.3-point (full-sample 95% CI: 0.4, 2.3; confirmatory p = 0.030) increase in BASC-2 externalizing score associated with a phthalate mixture heavily weighted for MCPP and MBzP. Other coefficients such as those for the suggestive mixture associations with worse adaptive skills did not appear to differ by sex. IPW had little effect on any model estimates (see Fig. A.8).

## 4. Discussion

### 4.1. Summary of findings and significance

Phthalate mixtures were significantly associated with adverse outcomes for many behavioral measures in the original models, and after applying the permutation tests, we have the highest confidence in the early pregnancy associations with autistic traits in both sexes, as well as the male-specific late pregnancy association with externalizing behaviors. The magnitudes of observed associations were all around 1 point per 1 quintile simultaneous increase in each component of the mixture, which is a substantial effect size considering that each T score unit can be interpreted as 0.1 SD of the population distribution. MBzP and MCPP were important metabolites in almost all observed associations, while sex-stratified analyses suggested that early pregnancy phthalate mixture associations specific to girls were predominantly driven by low molecular weight phthalates (LMWP) like DEP and DBP metabolites, while higher molecular weight phthalates like DEHP metabolites were only important in some male-specific associations. The mixture associations we evaluated more accurately model real-world, ubiquitous phthalate co-exposures than the conventional approach of evaluating individual phthalates.

Our results suggest significant relationships between everyday ambient exposures and adverse child behavior and therefore have substantial implications for neurobehavioral development. Although associations were relatively modest, and most children in this low-risk cohort scored within the average range relative to BASC-2 and SRS-2 test norms, the clinical significance of our findings is underscored by the widespread exposure to phthalates in the environment. From a public health perspective, even a small association between phthalates and neurodevelopment may have profound implications for the general population (Bellinger 2012). While legislative efforts have reduced exposure to some of these compounds (e.g., DEP, DBP, BBzP, DEHP), others have significantly increased (e.g., DiBP, DiNP) (Zota et al. 2014). Furthermore, exposure may be much higher in vulnerable child populations (e.g., neonates in intensive care units), with the potential to compound other neurodevelopmental risk factors.

### 4.2. Potential mechanisms of neurobehavioral effects of phthalate mixtures

This study did not assess potential mechanisms underlying the associations between phthalate mixtures and neurobehavioral outcomes. However, research in animal models and a few human studies provide mechanistic hypotheses to inform future research. Phthalates are known to inhibit the synthesis of testosterone (Wilson et al. 2008), and our group and others have observed that exposure to phthalates in pregnancy affects human maternal sex hormone concentrations (Lin et al. 2011; Sathyanarayana et al. 2017). Sex hormone concentrations may, in turn, affect child neurobehavioral outcomes. For example, prenatal androgen concentrations are associated with conditions like attention deficit hyperactivity disorder (ADHD) and autism in humans (Martel 2013). Data regarding estrogen are limited, though animal studies suggest that prenatal estrogen may be associated with anxiety and behavioral inhibition (Schulz and Sisk 2016). Perhaps our observed suggestive associations between early pregnancy mixtures highly weighted for LMWP and externalizing and adaptive skills were specific to girls due to their vulnerability to increased estrogen signaling, as suggested by female-specific associations between the estrogenic compound bisphenol A (BPA) and childhood BASC-2 externalizing scores that were stronger when evaluating early pregnancy (Braun et al. 2009). The potential mechanisms underlying the late pregnancy male-specific phthalate associations with externalizing behaviors we observed are less clear, though other epidemiologic studies have demonstrated male-specific associations between gestational phthalates and externalizing behaviors (Engel et al. 2010; Kobrosly et al. 2014).

Alternatively, multiple phthalates have been shown in vitro to competitively inhibit the thyroid receptor (Ghisari and Bonefeld-Jorgensen 2009). In another pregnancy cohort, WQS regression mixture analyses suggested that an early second trimester phthalate mixture with high weights for MEP and MCPP was associated with lower total thyroxine in maternal serum (Romano et al. 2018), which in early gestation has been associated with faulty neuronal migration in rodents as well as with higher age 6 SRS scores, which may underlie our observed SRS-2 score associations specific to the early pregnancy period (Roman et al. 2013). Accordingly, a recent study suggested impaired white matter development may mediate mid-pregnancy phthalate associations with child behavior (England-Mason et al. 2020a). These mechanisms are not mutually exclusive, and phthalates may in fact act through multiple pathways to exert cumulative additive effects (Howdeshell et al. 2008a; Howdeshell et al. 2008b; Rider et al. 2008).

### 4.3. Comparing mixture results to individual phthalate model results in the literature

Our data suggest that the associations between phthalates and neurobehavioral outcomes may be sex and time-specific. DEHP metabolites were moderately or highly weighted only for significant mixture associations in boys; MEP, MBP, and MiBP weights were only highly weighted for significant associations in girls; and MBzP and MCPP were highly weighted for most associations. A recent study also used WQS regressions to examine associations between age 7 Conner’s Parent Rating Scale (CPRS) scores and mixtures of 8 phthalate metabolites measured in the third trimester and at child ages 3 and 5, finding no significant mixture associations when evaluating all measured phthalates (Daniel et al. 2020). However, when restricting only to the mixture of DEHP metabolites, this study observed prenatal phthalate mixture associations with increased odds of above-median social problems in males as well as anxiety and emotional liability symptoms in females, in addition to an age 3 mixture association with cognitive problems in girls and an age 5 mixture association with emotional liability in both sexes. These findings differ from ours likely due to the different behavior outcomes, the lack of evaluation of multiple gestational time points, the more limited sample size and set of confounders, the likely reduction in statistical power incurred by dichotomizing continuous outcomes (Altman and Royston 2006), and the low power and unstable estimates inherent in single-split WQS regressions (Loftus et al. 2020). Another recent study also utilized WQS regression to examine associations between age 8 BASC-2 outcomes and cumulative exposure to 11 phthalate metabolites measured at several postnatal timepoints between ages 1–8, finding significant mixture associations with externalizing, internalizing, and BSI scores with high weights for MBzP, monocarboxynonyl phthalate (MCNP), and MEP for all associations, with associations remaining consistent when adjusting for exposure to lead and other cocontaminants (Li et al. 2020). This study focused on postnatal mixture associations rather than assessing critical gestational windows or sex-specific mixture effects as our study did, which may explain differences in our observed BASC-2 results, though we also observed a consistently important contribution of MBzP to mixture associations with BASC-2 outcomes and individual metabolite regressions separately evaluating the gestational period in that study also observed significant associations of MCPP with each outcome, similar to our gestational mixture results.

Regarding studies that have analyzed phthalate metabolites individually, a recent study observed early pregnancy MBP and MCPP positive associations with age 3–4 SRS-2 total score, similar to our findings, though only in boys with inadequate folic acid supplementation, which we did not measure (Oulhote et al. 2020). Furthermore; a recent paper found that age 3–4 BASC-2 externalizing score and/or BSI associations with second trimester MBzP, MiBP, MBP, and the sum of LMWP that tended to be stronger in males were not detected when evaluating analogous CBCL scores; with the authors suggesting that the BASC-2 may be more sensitive in detecting subclinical changes in behavior (England-Mason et al. 2020b). We also observed an important contribution of MBzP in sex-specific mixture associations with internalizing behavior, but in our analysis the contribution of MBP and MiBP was specific to females in early pregnancy. Our findings of the importance of MCPP in most observed associations is supported by longitudinal age 7–16 BASC-2 externalizing and internalizing assessment associations with MCPP recently observed (Hyland et al. 2019). Similar to our suggestive late pregnancy mixture trends in both sexes; other studies have also observed late pregnancy DEHP metabolite associations with age 2–14 CBCL or Behavior-Style Questionnaire (BSQ) adaptability and externalizing, internalizing, and BSI-related behaviors, though without observing sex-specificity (Huang et al. 2019; Ku et al. 2019; Lien et al. 2015). However, several other studies found null associations between age 1–5 SRS, CBCL, and Strengths and Difficulties Questionnaire (SDQ) behavior outcomes and DEHP metabolites (Braun et al. 2014; Kim et al. 2018; Philippat et al. 2017; Whyatt et al. 2012). Previous studies have also observed LMWP and MEP associations with BASC or CBCL externalizing behaviors or SRS autistic traits between ages 3–9 (Engel et al. 2010; Lien et al. 2015; Miodovnik et al. 2011) and MBP associations with BASC, CBCL, SDQ, or BSQ externalizing behaviors, withdrawal, and adaptability between ages 2–11; though without the sex- and timespecificity we observed (Engel et al. 2010; Kobrosly et al. 2014; Ku et al. 2019; Lien et al. 2015; Philippat et al. 2017; Whyatt et al. 2012). Contrary to our findings, MBP has been associated with age 3–9 BASC or CBCL internalizing behaviors (Engel et al. 2010; Lien et al. 2015; Miodovnik et al. 2011; Whyatt et al. 2012); MBzP has been associated with age 2–14 CBCL, SDQ, or BSQ internalizing or BSI-related behaviors in late pregnancy (Huang et al. 2019; Ku et al. 2019; Philippat et al. 2017; Whyatt et al. 2012); MCPP has been negatively associated with odds of age 3 autism spectrum disorder (ASD) diagnosis (Shin et al. 2018); and MEP has been associated with age 1–9 CBCL or SDQ internalizing behaviors and BASC BSI (Engel et al. 2010; Jankowska et al. 2019; Kim et al. 2018). Other studies have even shown protective associations between MEP or DEHP metabolites in girls or both sexes and age 1–10 CPRS or CBCL internalizing or ADHD symptoms (Gascon et al. 2015;Kobrosly et al. 2014). Variation in neurobehavioral effects as a function of sex, timing of exposure and outcome assessment, and individual phthalates and behaviors studied highlights the complexity of this research and the need for future studies of effect modification and simultaneous analysis of phthalate mixtures to more closely resemble real-world exposure.

### 4.4. Strengths and limitations

Strengths of this study include the measurement of several phthalate metabolites across multiple time points during pregnancy; a large sample of healthy pregnancies; the assessment of both behavioral problems and adaptive behaviors using standardized, norm-referenced measures; consideration of effect modification by sex; adjustment for important predictors of child behavior outcomes; and a modern statistical approach to analyze mixture exposure associations with outcomes. This study has several key limitations. Spot urine samples may be influenced by random variation in daily concentrations due to acute exposures. We attempted to reduce that variation by curvilinearly controlling for the time of day of urine collection, which should improve estimate precision. If this variation is similarly random across pregnancy periods, this should not bias our estimates. Our reliance on spot urine samples at only two different time points will increase the chances of results being affected by exposure misclassification, and so our timing-specific results should be interpreted with caution until further verification. It is possible that differences in WQS mixture weights for individual metabolites across time may be in part due to differences in concentrations over time, but this is unlikely to be a major determinant of the observed timing-specific mixture weights because weights do not consistently change in the same direction as concentrations between the time periods. The TIDES cohort is also limited in that it is mainly comprised of higher income, predominantly white, well-educated families, which limits the generalizability of these findings. In addition, participants without complete data excluded from this analysis were more likely to be black, less well-educated, and have lower incomes, though IPW analyses suggest that this had minimal influence on effect estimates. The WQS regression method is limited to evaluating additive, monotonic associations between mixture exposures and outcomes, and so we did not examine synergistic, antagonistic, or non-monotonic associations between phthalate mixtures and behavioral outcomes. Mixture association effect sizes should be interpreted keeping in mind that within each time period, correlations for some phthalates were relatively low in our population, so a simultaneous quantile increase in each phthalate metabolite may be uncommon. However, this may not be the case in other populations, and the mixture weights combined with the mixture association effect size provide information on estimating effect sizes for increases of one or more components out of the total mixture.

## 5. Conclusions

After analyzing the mixture effects of maternal phthalate concentrations on child behavioral outcomes, we observed adverse mixture associations with many measured behavior scores, in particular significant associations with autistic traits and externalizing behaviors. These results suggest cumulative, additive impacts of phthalate mixtures, with the most important phthalates in that mixture differing by outcome, sex, and timing of exposure.

## Supplementary Material

Supplementary Material

## Figures and Tables

**Table 1:** Demographic Characteristics of 501 Mother-Child Dyads in the TIDES Study with Complete Maternal Phthalate, Child Neurobehavioral Outcome, and Covariate Data for at Least One Trimester.

Characteristic	Mean (SD)	Median (Range)
Maternal age (years)	31.61 (5.31)	31.98 (18.25 – 44.26)
Pre-pregnancy BMI	25.56 (6.32)	23.49 (16.92 – 59.39)
Birth weight (kg)	3.34 (0.57)	3.32 (0.55 – 5.15)
Gestational age at birth (weeks)	39.22 (1.89)	39.57 (25.00 – 42.43)
Child age (years)	4.53 (0.34)	4.54 (3.93 – 5.95)
Maternal PHQ-9 Score	3.13 (3.71)	2 (0 – 23)
Maternal PSS-10 Score	14.26 (5.96)	14 (1 – 33)
Gestational age at urine collection:		
Early pregnancy (weeks)	10.74 (2.08)	11.00 (5.14 – 20.43)
Late pregnancy (weeks)	32.60 (3.01)	32.00 (25.71 – 41.14)
Time of day of urine collection:		
Early pregnancy	12:19 (2.49 hrs)	12:00 (07:30 – 19:50)
Late pregnancy	12:02 (2.70 hrs)	11:15 (07:45 – 18:00)
Characteristic	N (%)	
Child Sex		
M	243 (48.50%)	
F	258 (51.50%)	
Study Center		
UCSF	142 (28.34%)	
UMN	142 (28.34%)	
URMC	126 (25.15%)	
UW	91 (18.16%)	
Race		
Asian	29 (5.59%)	
Black	51 (10.18%)	
Other	59 (11.78%)	
White	363 (72.46%)	
Income		
≤ $25,000	101 (20.16%)	
$25,001 – $75,000	135 (26.95%)	
> $75,000	265 (52.89%)	
Education		
High School or less	59 (11.78%)	
College	205 (40.92%)	
Graduate School	237 (47.31%)	
Parity		
Primiparous	271 (54.09%)	
Multiparous	230 (45.91%)	
Alcohol Consumption During Pregnancy		
No	435 (86.83%)	
Yes	66 (13.17%)	
Cigarette Use During Pregnancy		
No	476 (95.01%)	
Yes	25 (4.99%)	

**Table 2:** Maternal Urinary Specific Gravity-Adjusted Phthalate Metabolite Concentrations (ng/mL) between Pregnancy Periods.

Phthalate	Period	N <LOD (%)^a^	Mean (SD)	Median (Range)	Difference^b^	p-value
MEP	Early	5 (1.0%)	104.0 (345.6)	30.0 (0.8 – 4293.0)	18.6%	0.03*
	Late	6 (1.3%)	291.4 (2135.3)	31.8 (1.6 – 43633.3)		
MBP	Early	42 (8.6%)	13.1 (27.0)	8.5 (0.4 – 432.1)	20.0%	5.8 × 10^−4^*
	Late	12 (2.6%)	23.5 (158)	10.7 (0.7 – 3373.3)		
MiBP	Early	17 (3.5%)	7.1 (7.3)	5.2 (0.2 – 57.7)	39.7%	2.9 × 10^−12^*
	Late	17 (3.7%)	11.5 (19.3)	6.9 (0.4 – 220.5)		
MBzP	Early	68 (14.0%)	8.0 (17.7)	3.9 (0.3 – 318.5)	10.4%	0.08
	Late	21 (4.5%)	10.9 (35.8)	4.5 (0.2 – 711.7)		
MEHP	Early	162 (33.3%)	5.3 (22.1)	2.4 (0.2 – 352.8)	−19.0%	3.6 × 10^−4^*
	Late	117 (25.2%)	3.5 (7.5)	2.0 (0.3 – 100.9)		
MEOHP	Early	15 (3.1%)	11.0 (39.0)	5.3 (0.2 – 679.3)	−5.4%	0.36
	Late	3 (0.6%)	9.0 (19.5)	5.4 (0.1 – 338.8)		
MEHHP	Early	15 (3.1%)	17.2 (66.3)	7.5 (0.2 – 1077.5)	−14.5%	0.01*
	Late	3 (0.6%)	11.8 (26.8)	6.9 (0.2 – 466.2)		
MECPP	Early	15 (3.1%)	19.4 (47.9)	9.3 (0.8 – 635.8)	13.3%	0.02*
	Late	0 (0%)	18.6 (36.5)	10.9 (0.7 – 555.8)		
MCPP	Early	133 (27.4%)	8.0 (42.3)	2.0 (0.1 – 802.9)	−5.2%	0.48
	Late	63 (13.5%)	7.0 (20.8)	1.8 (0.1 – 266.8)		

a Values are shown for 486 observations in early pregnancy and 464 observations in late pregnancy with complete outcome and covariate data.

b Paired t-tests evaluated differences in mean concentration between the late minus the early pregnancy periods for 448 participants with complete data for both time periods. Phthalates were log_10_-transformed prior to analysis with the paired *t*-test due to right skew, and mean differences in log_10_ values are presented as percent differences for interpretability.

**Table 3:** BASC-2 and SRS-2 Composite T Scores between Male and Female Children.

Variable	Group	N	Mean (SD)	Median (Range)	t-test p-value^a^
BASC EXT	Total	500	49.65 (8.45)	48 (33 – 110)	0.41
	M	243	49.33 (7.88)	48 (33 – 73)	
	F	257	49.96 (8.97)	48 (34 – 110)	
BASC INT	Total	500	48.87 (8.97)	48 (30 – 82)	0.01*
	M	243	47.82 (8.73)	47 (30 – 77)	
	F	257	49.86 (9.1)	49 (31 – 82)	
BASC ADS	Total	500	50.77 (8.55)	51 (20 – 72)	0.38
	M	243	51.12 (8.82)	52 (20 – 70)	
	F	257	50.45 (8.29)	51 (25 – 72)	
SRS TOTAL	Total	501	45.09 (6.91)	44 (34 – 81)	0.01*
	M	243	45.93 (7.01)	45 (35 – 74)	
	F	258	44.30 (6.73)	43 (34 – 81)	

a t-tests evaluated differences in BASC-2 and SRS-2 composite T scores between female and male children. EXT = externalizing, INT = internalizing, and ADS = adaptive skills. Clinically relevant T scores are considered to be those above 60 for all scores except for adaptive skills, for which clinically relevant scores would be below 40.

## Abbreviations:

ADS: adaptive skills score
BASC-2: Behavioral Assessment System for Children, 2nd Edition
BSI: behavioral symptoms index score
EXT: externalizing behavior score
INT: internalizing behavior score
SRS-2: Social Responsiveness Scale, 2nd Edition
WQS: weighted quantile sum
