# Supplementary material for: Phthalate mixtures in pregnancy, autistic traits, and adverse childhood behavioral outcomes
Source: Environ Int. Author manuscript; Available in PMC 2022 Jul 18. (PMC9291724; doi:10.1016/j.envint.2020.106330)
Supplement: Supplementary Material [file NIHMS1822467-supplement-Supplementary_Material.docx]

**Supplemental Material**

**Phthalate Mixtures in Pregnancy, Autistic Traits, and Adverse Childhood Behavioral Outcomes**

Drew B. Day, Brent R Collett, Emily S Barrett, Nicole R Bush, Shanna H Swan, Ruby HN Nguyen, Adam A Szpiro, and Sheela Sathyanarayana

**Appendix A**

**Table of Contents**

**Results A.1. Summary Data and Information on Phthalate Metabolites**

**Table A.1.** Phthalate Metabolite Analytes and Associated Parent Compounds

**Table A.2.** Phthalate Metabolite Limits of Detection (LODs) by Pregnancy Period, Lab, and Batch

**Figure A.1.** Phthalate Metabolite Concentrations and Quintile Break Points by Study Center and Sex

**Results A.2. BASC and SRS Score Distributions**

Figure A.2: BASC and SRS Composite Score Distributions by Sex

**Results A.3. Pearson Correlations**

**Figure A.3.** Pearson Correlations Between Early Pregnancy Phthalate Measurements

**Figure A.4.** Pearson Correlations Between Late Pregnancy Phthalate Measurements

**Figure A.5.** Pearson Correlations Between Early and Late Pregnancy Phthalate Measurements

**Figure A.6.** Pearson Correlations Between Behavior Assessment Composite Scores

**Results A.4. IPW Methods and Results**

**Table A.3.** Demographic Characteristics Compared between Participants Included and Excluded from the Final Analysis

Figure A.7. WQS Regression Coefficients and Weights for Associations between Phthalate Mixtures in Early and Late Pregnancy and Behavior in the Total Population using Inverse Probability Weighting

Figure A.8. WQS Regression Coefficients and Weights for Associations between Phthalate Mixtures in Early and Late Pregnancy and Behavior Stratified by Sex Using Inverse Probability Weighting

**Results A.5. Model Results for BASC-2 BSI**

**Table A.4.** BASC-2 BSI Composite T Scores between Male and Female Children

Figure A.9. All WQS Regression Coefficients and Weights for Associations between Phthalate Mixtures in Early and Late Pregnancy and BASC-2 BSI Either in Models Evaluating the Total Population or in Models Stratifying by Sex and Either Not Using Inverse Probability Weighting (IPW) or Using IPW

**Results A.6. WQS Regression Coefficients**

**Table A.5.** WQS Regression Mixture Coefficient Results

**Results A.7. Individual Phthalate Linear Quintile Regression Coefficients**

Figure A.10. All Individual Phthalate Regression Coefficient Means and 95% CIs in either Early or Late Pregnancy for the Total Study Population

**Figure A.11.** All Female-Specific Sex-Stratified Individual Phthalate Regression Coefficient Means and 95% CIs in either Early or Late Pregnancy

**Figure A.12.** All Male-Specific Sex-Stratified Individual Phthalate Regression Coefficient Means and 95% CIs in either Early or Late Pregnancy

**Results A.1. Summary Data and Information on Phthalate Metabolites**

Table A.1 shows each of the urinary phthalate metabolites analyzed in this study, and displays their molecular weights and associated parent compounds.

Table A.1. Phthalate Metabolite Analytes and Associated Parent Compounds

| **Metabolite (molecular weight (g/mol))** | **Parent compound(s) (molecular weight (g/mol))** |
| --- | --- |
| Monoethyl phthalate (MEP) (194) | Diethyl phthalate (DEP) (222) |
| Mono-n-butyl phthalate (MBP) (222) | Major: Dibutyl phthalate (DBP) (278)  Minor: Butylbenzyl phthalate (BBzP) (312) |
| Monoisobutyl phthalate (MiBP) (222) | Diisobutyl phthalate (DiBP) (278) |
| Monobenzyl phthalate (MBzP) (256) | Butylbenzyl phthalate (BBzP) (312) |
| Mono(2-ethylhexyl) phthalate (MEHP) (278) | Di-2-ethylhexyl phthalate (DEHP) (390) |
| Mono(2-ethyloxohexyl) phthalate (MEOHP) (292) |  |
| Mono(2-ethyl-5-hydroxyhexyl) phthalate (MEHHP) (294) |  |
| Mono(2-ethyl-5-carboxypentyl) phthalate (MECPP) (308) |  |
| Mono(3-carboxypropyl) phthalate (MCPP) (252) | Major: Di-n-octyl phthalate (DnOP) (394)  Minor: Dibutyl phthalate (DBP) (278)  Minor: Other high molecular weight phthalates |

Table A.2 shows the limit of detection (LOD) values for each phthalate metabolite separated by pregnancy period and laboratory and batch at which each sample was measured. Samples were analyzed either at the Environmental Health Laboratory at the University of Washington (UW Lab), or at the Division of Laboratory Sciences, National Center for Environmental Health, Centers for Disease Control and Prevention (CDC Lab) in one of three batches with at times varying LODs.

Table A.2. Phthalate Metabolite Limits of Detection (LODs) by Pregnancy Period, Lab, and Batch

| Phthalate Metabolite | LOD (ng/mL) (N samples below LOD) | | | |
| --- | --- | --- | --- | --- |
|  | Early Pregnancy (N = 486) | | Late Pregnancy (N = 464) | |
|  | UW Lab (N = 189 (38.9%)) | CDC Lab Batch 1 (N = 297 (61.1%)) | CDC Lab Batch 2 (N = 108 (23.3%)) | CDC Lab Batch 3 (N = 356 (76.7%)) |
| MEP | 1.0 (2) | 0.6 (3) | 1.2 (0) | 1.2 (6) |
| MBP | 1.0 (16) or 2.0 (6) | 0.4 (20) | 0.4 (5) | 0.4 (7) |
| MiBP | 0.2 (5) or 1.0 (3) | 0.2 (9) | 0.2 (1) | 0.8 (16) |
| MBzP | 1.0 (43) | 0.3 (25) | 0.3 (4) | 0.3 (17) |
| MEHP | 1.0 (70) | 0.5 (78) | 0.5 (35) | 0.8 (82) |
| MEOHP | 1.0 (9) | 0.2 (6) | 0.2 (1) | 0.2 (2) |
| MEHHP | 1.0 (7) | 0.2 (2) | 0.4 (0) | 0.4 (3) |
| MECPP | 1.0 (4) | 0.2 (0) | 0.4 (0) | 0.4 (0) |
| MCPP | 1.0 (97) | 0.2 (34) | 0.2 (10) | 0.4 (53) |

Figure A.1 shows how maternal urinary phthalate concentrations varied across study centers, pregnancy time periods, and between the stratified datasets. The colored bands represent the four quintile cutpoints for each study center-specific quintile and show how they varied across these parameters as well. The variability and medians were relatively similar across study centers for many metabolites, particularly the DEHP metabolites, but there was more heterogeneity by study center for certain metabolites, particularly MEP, MBzP, and MCPP. This may reflect regional, socioeconomic, or cultural differences in personal care product use or in other major sources of phthalate exposure. In each of these cases, the URMC center had the highest concentrations and often the greatest range of concentrations. The use of study center-specific quintiles allowed for this heterogeneity to be controlled in the models. We expect exposures, outcomes, and many measured and unmeasured covariates to vary by study center, and so we chose study center-specific quintiles to focus more on within-center differences in phthalate levels rather than focusing on comparability across study centers.

Figure A.1 Maternal Urinary Phthalate Metabolite Concentrations and Quintile Break Points by Study Center and Child Sex


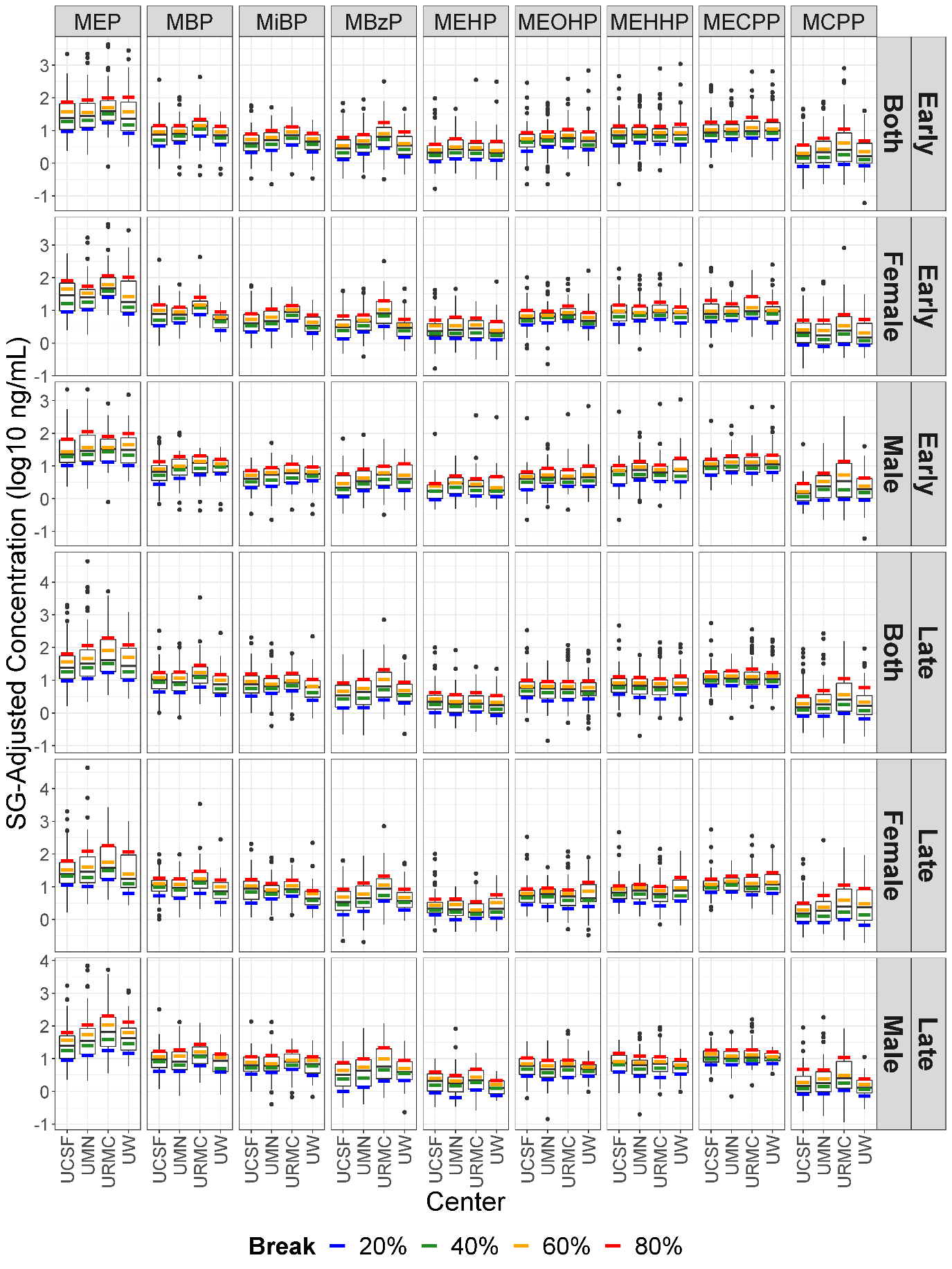


**Results A.2. BASC and SRS Score Distributions**

Figure A.2 shows boxplots stratified by sex with overlaid points denoting the means for all BASC and SRS composite scores.

Figure A.2: BASC and SRS Composite Score Distributions by Sex


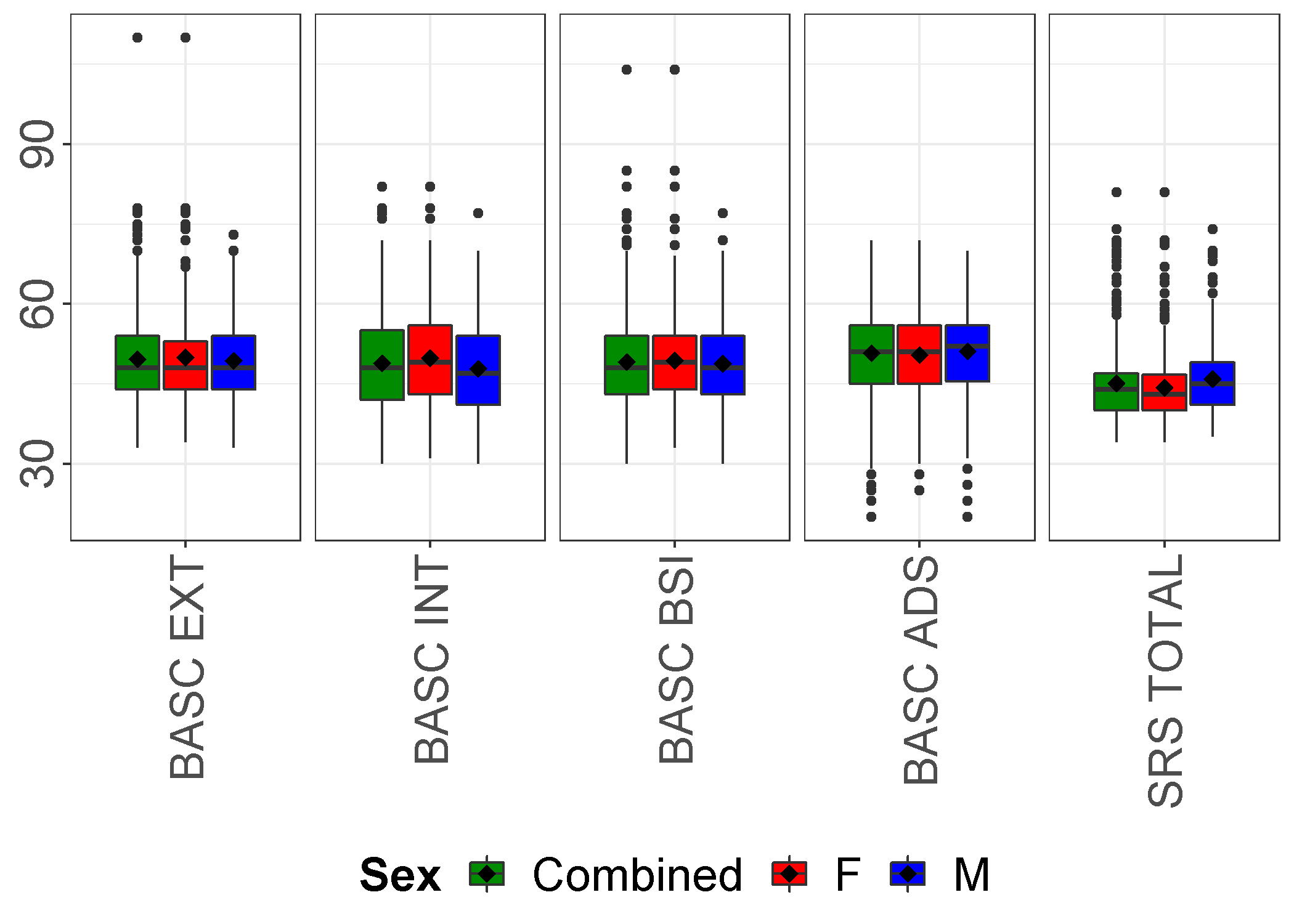


**Results A.3. Pearson Correlations**

The following plots show Pearson correlations for exposure and outcome measures in this study for the 501 participants with complete data for all covariates, all phthalate metabolites in at least one of the two pregnancy periods, and at least one of the five behavior composite scores. Figure A.3 shows Pearson correlations between log_10_-transformed, specific gravity-adjusted phthalates measured in early pregnancy, while Figure A.4 shows Pearson correlations between log_10_-tranformed, specific gravity-adjusted phthalates in late pregnancy. Figure A.5 shows Pearson correlations between log_10_-transformed, specific gravity-adjusted phthalates measured in early (x-axis) and late (y-axis) pregnancy. Correlations for the same metabolite between the early and late pregnancy periods can be found on the diagonal. Figure A.6 shows Pearson correlations between BASC-2 and SRS-2 composite scores.

Figure A.3. Pearson Correlations Between Early Pregnancy Phthalate Measurements


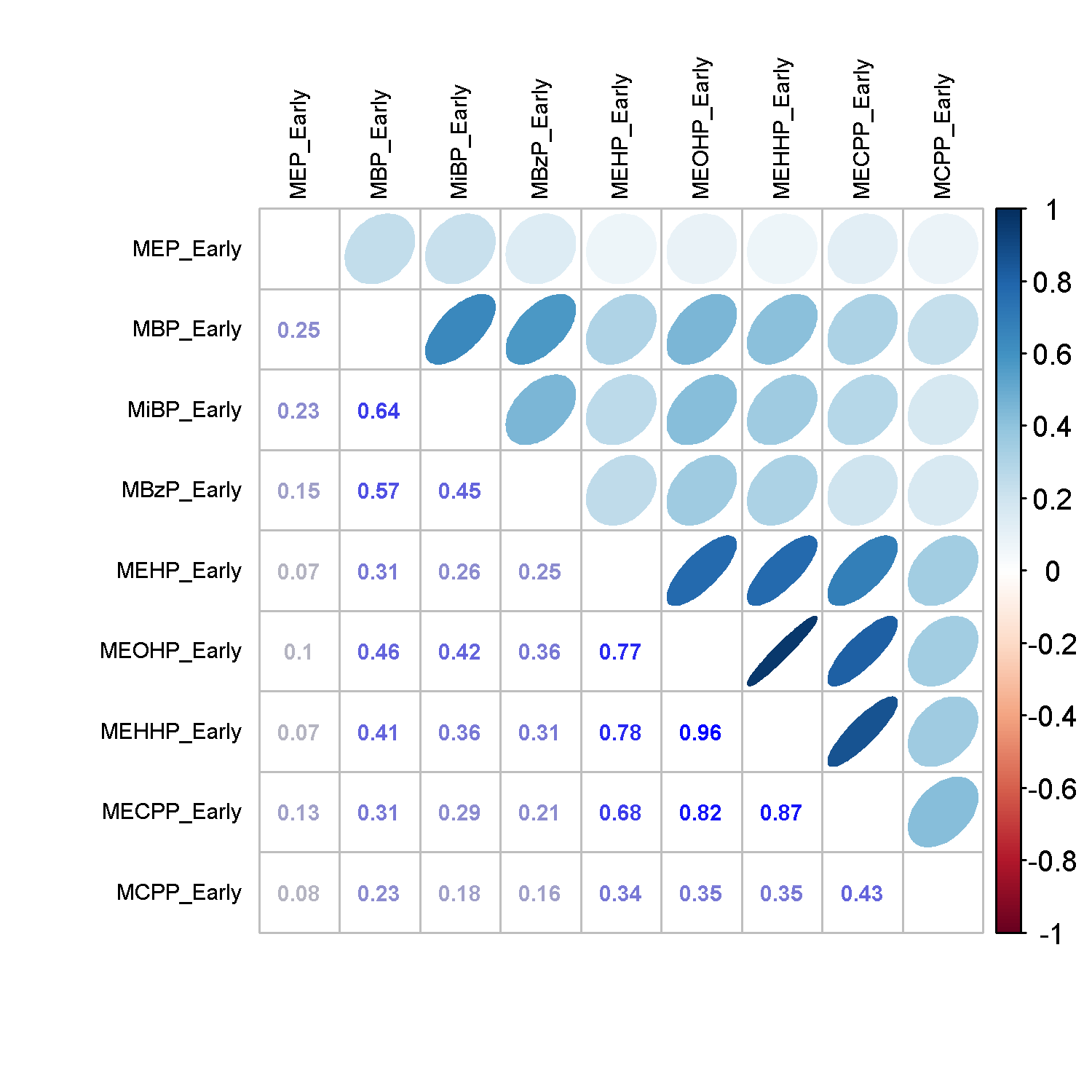


Figure A.4. Pearson Correlations Between Late Pregnancy Phthalate Measurements


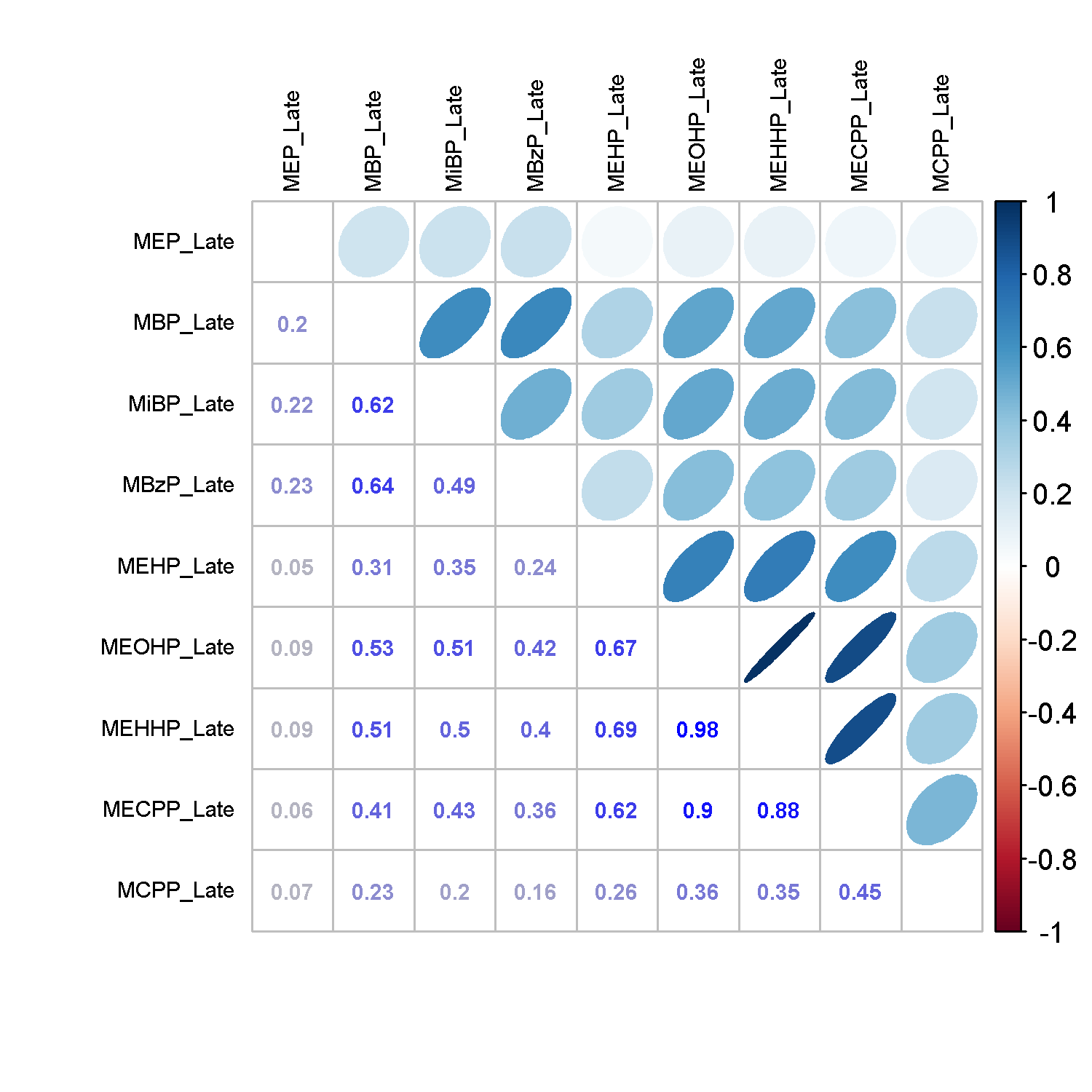


Figure A.5. Pearson Correlations Between Early and Late Pregnancy Phthalate Measurements


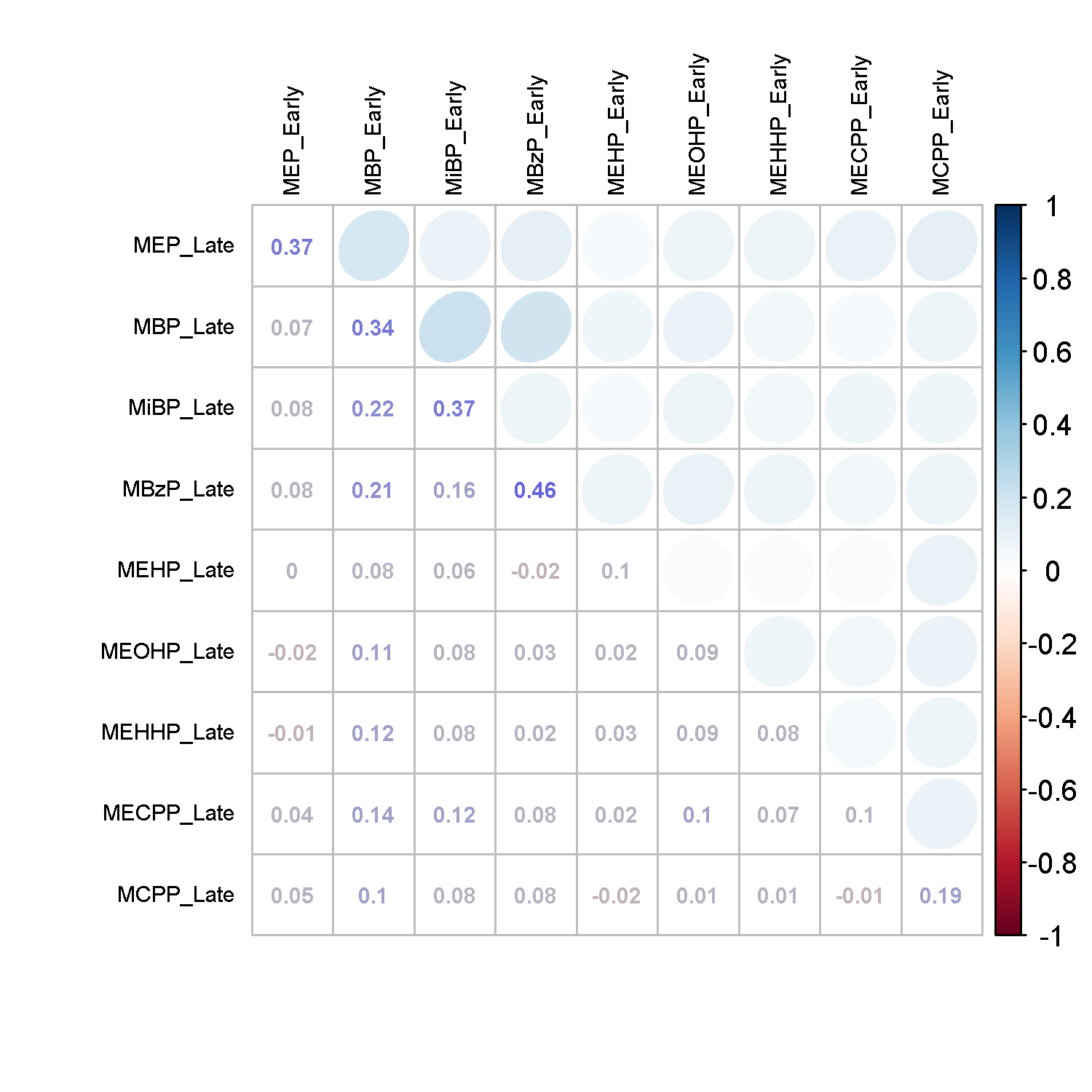


Figure A.6. Pearson Correlations Between Behavior Assessment Composite Scores


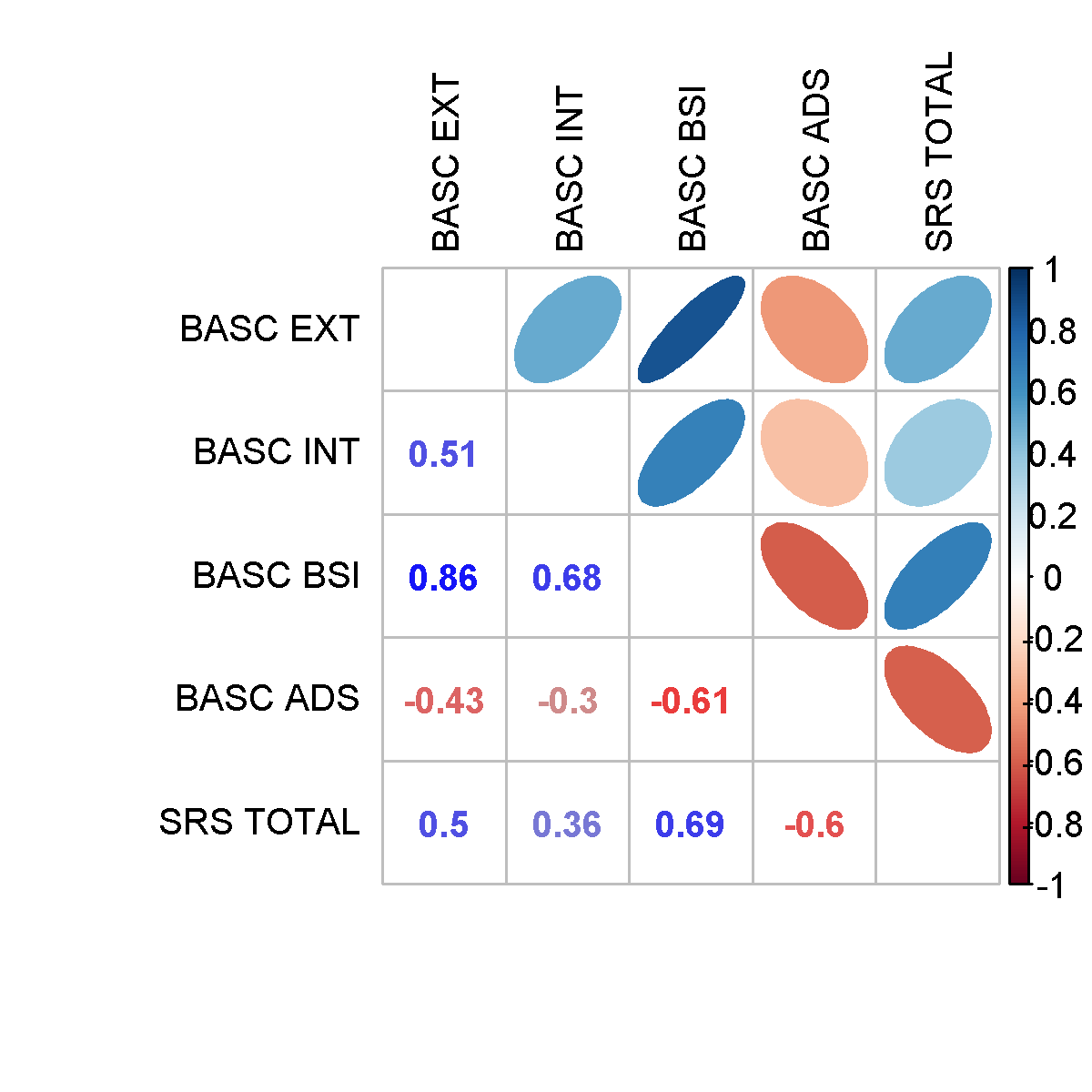


**Results A.4. IPW Methods and Results**

In order to account for potential bias introduced by differential demographic characteristics between participants included in the final analysis (n = 500 mothers and 501 children) and those lost to follow-up or otherwise excluded due to incomplete data (n = 301 mothers and children), we generated inverse probability weights to weight each observation by the inverse probability of inclusion (Seaman and White 2013). We used a logistic regression to regress a dichotomous variable of inclusion in the final analysis on the a priori-selected demographic variables thought to be potentially related to inclusion probability, namely study center, race, income, education, dichotomous parity, household population, maternal age, child sex, and gestational age at birth. The values for these variables are compared between the included and excluded participant groups in Table A.3. Significant coefficients for the logistic regression included being of the race “other” (OR = 2.6 (95% CI: 1.2, 5.7)) and gestational age at birth (OR = 0.9 (0.8, 0.99)). Inverse probability weights were generated based on the predicted probability of inclusion for each participant, with weights equaling the inverse of that predicted probability for included participants and the inverse of one minus that predicted probability for excluded participants. These weights were then applied to the WQS regressions by including them in the “weights” input of the gwqs function in R and otherwise using the same methods as the WQS regressions without the weights. The results for WQS regressions excluding or including a WQS by sex interaction term are shown in Figures A.7 and A.8, respectively. Details on how to interpret these figures can be found in the Figure 1 and 2 captions in the main text.

Table A.3. Demographic Characteristics Compared between Participants Included and Excluded from the Final Analysis

| **Characteristic** | **Included** | **N** | **Mean (SD)** | **Median (Range)** | **t-test**  **p-value^a^** |
| --- | --- | --- | --- | --- | --- |
| Maternal Age (y) | N | 298 | 30.15 (5.88) | 30.48 (18.15 - 45.16) | 0.00050* |
|  | Y | 501 | 31.61 (5.31) | 31.98 (18.25 - 44.26) |  |
| Gestational Age at | N | 301 | 39.34 (1.91) | 39.57 (26 - 42.14) | 0.42 |
| Birth (weeks) | Y | 501 | 39.22 (1.89) | 39.57 (25 - 42.43) |  |
| Household | N | 292 | 2.72 (1.52) | 2 (0 - 11) | 0.61 |
| Population | Y | 501 | 2.67 (1.13) | 2 (0 - 10) |  |

| **Characteristic** | **Group** | **Excluded** | **Included** | **χ^2^ p-value^b^** |
| --- | --- | --- | --- | --- |
| Child Sex | M | 150 (38.07%) | 244 (61.93%) | 0.78 |
|  | F | 150 (36.86%) | 257 (63.14%) |  |
| Study Center: UCSF | No | 246 (40.66%) | 359 (59.34%) | 0.0018* |
|  | Yes | 55 (27.92%) | 142 (72.08%) |  |
| Study Center: UMN | No | 230 (39.05%) | 359 (60.95%) | 0.16 |
|  | Yes | 71 (33.33%) | 142 (66.67%) |  |
| Study Center: URMC | No | 202 (35.01%) | 375 (64.99%) | 0.022* |
|  | Yes | 99 (44.00%) | 126 (56.00%) |  |
| Study Center: UW | No | 225 (35.43%) | 410 (64.57%) | 0.021* |
|  | Yes | 76 (45.51%) | 91 (54.49%) |  |
| Race: Asian | No | 275 (36.76%) | 473 (63.24%) | 0.30 |
|  | Yes | 23 (45.10%) | 28 (54.90%) |  |
| Race: Black | No | 244 (35.16%) | 450 (64.84%) | 0.0019* |
|  | Yes | 54 (51.43%) | 51 (48.57%) |  |
| Race: Other | No | 260 (37.04%) | 442 (62.96%) | 0.77 |
|  | Yes | 38 (39.18%) | 59 (60.82%) |  |
| Race: White | No | 115 (45.45%) | 138 (54.55%) | 0.0015* |
|  | Yes | 183 (33.52%) | 363 (66.48%) |  |
| Income: <=$25000 | No | 189 (32.09%) | 400 (67.91%) | 0.0022* |
|  | Yes | 82 (44.81%) | 101 (55.19%) |  |
| Income: $25001-$75000 | No | 190 (34.17%) | 366 (65.83%) | 0.43 |
|  | Yes | 81 (37.50%) | 135 (62.50%) |  |
| Income: >$75000 | No | 163 (40.85%) | 236 (59.15%) | 0.00071* |
|  | Yes | 108 (28.95%) | 265 (71.05%) |  |
| Education: High School | No | 240 (35.19%) | 442 (64.81%) | 0.032* |
|  | Yes | 51 (46.36%) | 59 (53.64%) |  |
| Education: College | No | 151 (33.78%) | 296 (66.22%) | 0.058 |
|  | Yes | 140 (40.58%) | 205 (59.42%) |  |
| Education: Grad School | No | 191 (41.98%) | 264 (58.02%) | 0.00051* |
|  | Yes | 100 (29.67%) | 237 (70.33%) |  |
| Parity | Primiparous | 154 (36.24%) | 271 (63.76%) | 0.44 |
|  | Multiparous | 115 (33.33%) | 230 (66.67%) |  |

^a^t-tests were comparing means for continuous variables between included and excluded participants. ^b^χ^2^ tests were comparing frequencies of categorical variable categories between included and excluded participants.

Figure A.7. WQS Regression Coefficients and Weights for Associations between Phthalate Mixtures in Early and Late Pregnancy and Behavior in the Total Population using Inverse Probability Weighting


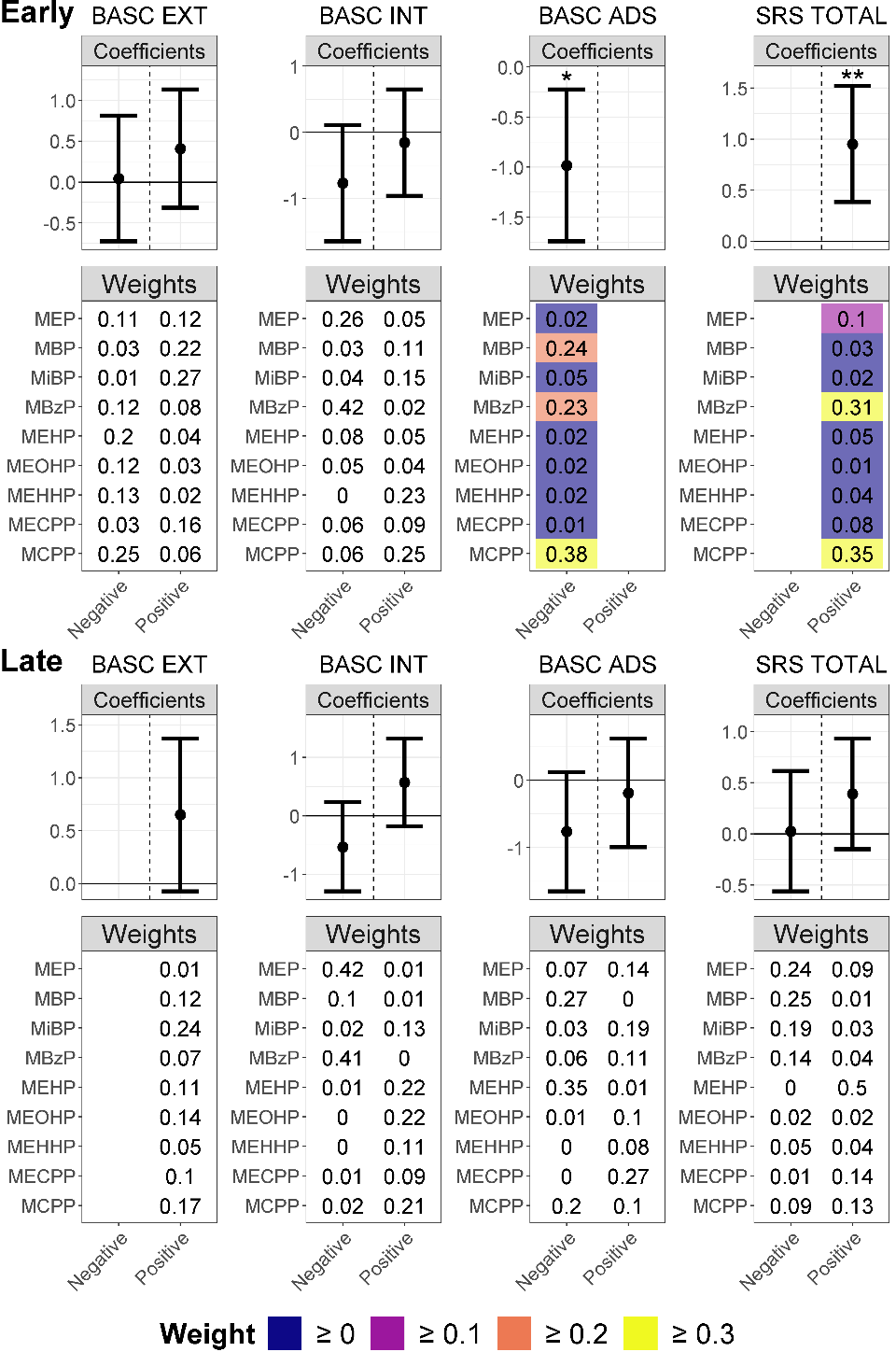


Figure A.8. WQS Regression Coefficients and Weights for Associations between Phthalate Mixtures in Early and Late Pregnancy and Behavior Stratified by Sex Using Inverse Probability Weighting


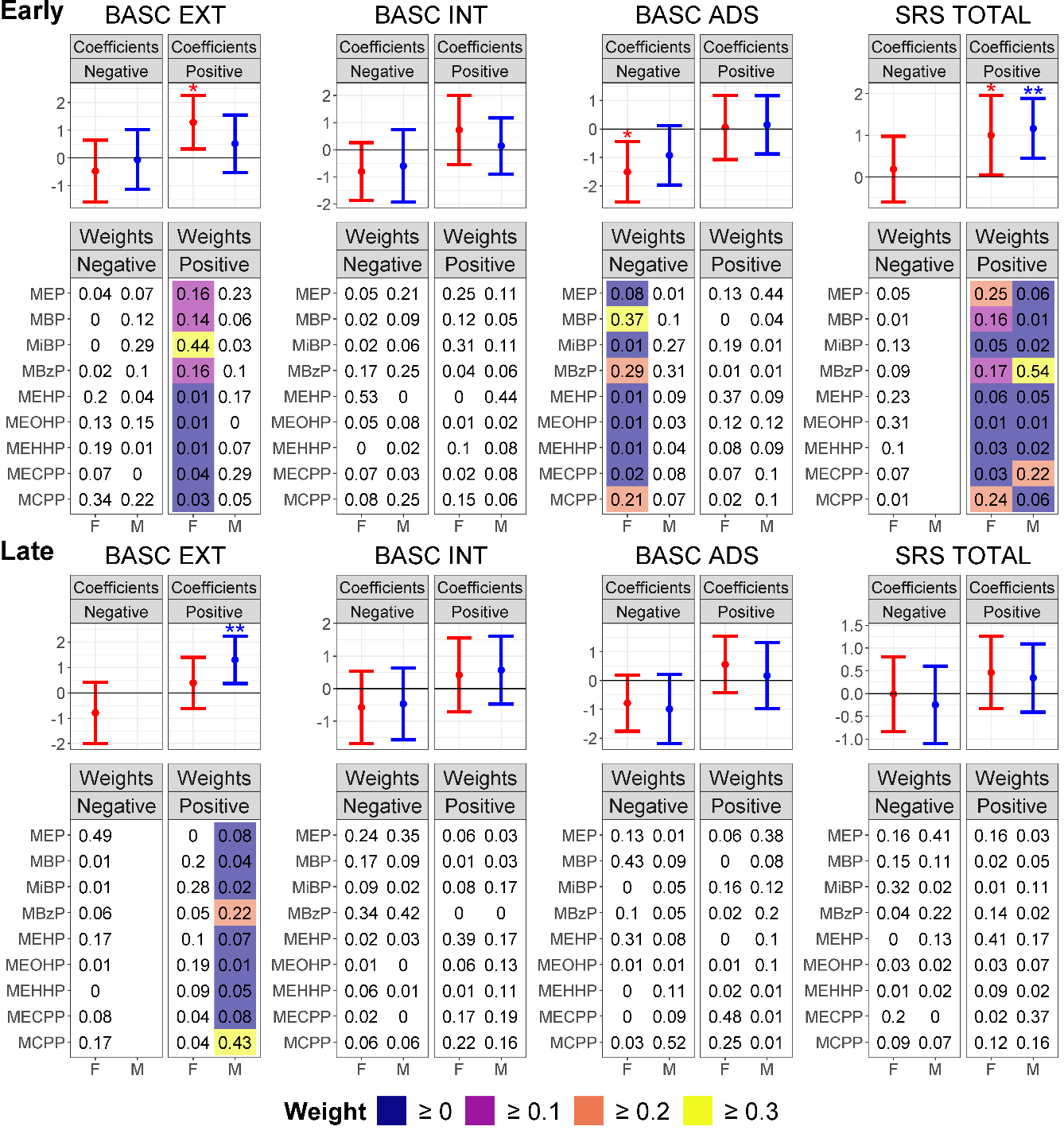


**Results A.5. Model Results for BASC-2 BSI**

BASC-2 BSI T scores are summarized in Table A.4, which shows that these scores were similar to normative sample distribution (mean = 50 and SD = 10) and did not differ between sexes.

Table A.4. BASC-2 BSI Composite T Scores between Male and Female Children

| **Variable** | **Group** | **N** | **Mean (SD)** | **Median (Range)** | **t-test p-value^a^** |
| --- | --- | --- | --- | --- | --- |
| BASC BSI | Total | 500 | 49.1 (8.64) | 48 (30 - 104) | 0.42 |
|  | M | 243 | 48.78 (8.2) | 48 (30 - 77) |  |
|  | F | 257 | 49.41 (9.04) | 49 (33 - 104) |  |

^a^t-tests evaluated differences in BASC-2 BSI composite T scores between female and male children. BSI = behavioral symptoms index.

All WQS regression results for models associating phthalate mixtures with BASC-2 BSI are shown in Figure A.9. Plots are arranged in two rows of four columns, with the top row showing results for early pregnancy phthalate mixtures (labeled “Early”) and the bottom row showing results for late pregnancy phthalate mixtures (labeled “Late”). The first two columns are the model results using the original data when evaluating both sexes without a sex interaction term (column 1; “Both Sexes”) and when evaluating WQS coefficients when stratifying the data by sex (column 2; “Sex-Stratified”). The latter two columns are the same as the first two except that these are the results with IPW. Details on how to interpret these figures can be found in the Figure 1 and 2 captions in the main text.

There are no significant associations between early or late pregnancy phthalate mixtures and BASC-2 BSI either considering the full-sample 95% CIs or the permutation test confirmatory p-value. There are however suggestive associations between an early pregnancy phthalate mixture and worse adaptive skills in girls, as well as between a late pregnancy phthalate mixture and increased externalizing behavior score in boys. Results were very similar with and without IPW.

Figure A.9. All WQS Regression Coefficients and Weights for Associations between Phthalate Mixtures in Early and Late Pregnancy and BASC-2 BSI Either in Models Evaluating the Total Population or in Models Stratifying by Sex and Either Not Using Inverse Probability Weighting (IPW) or Using IPW


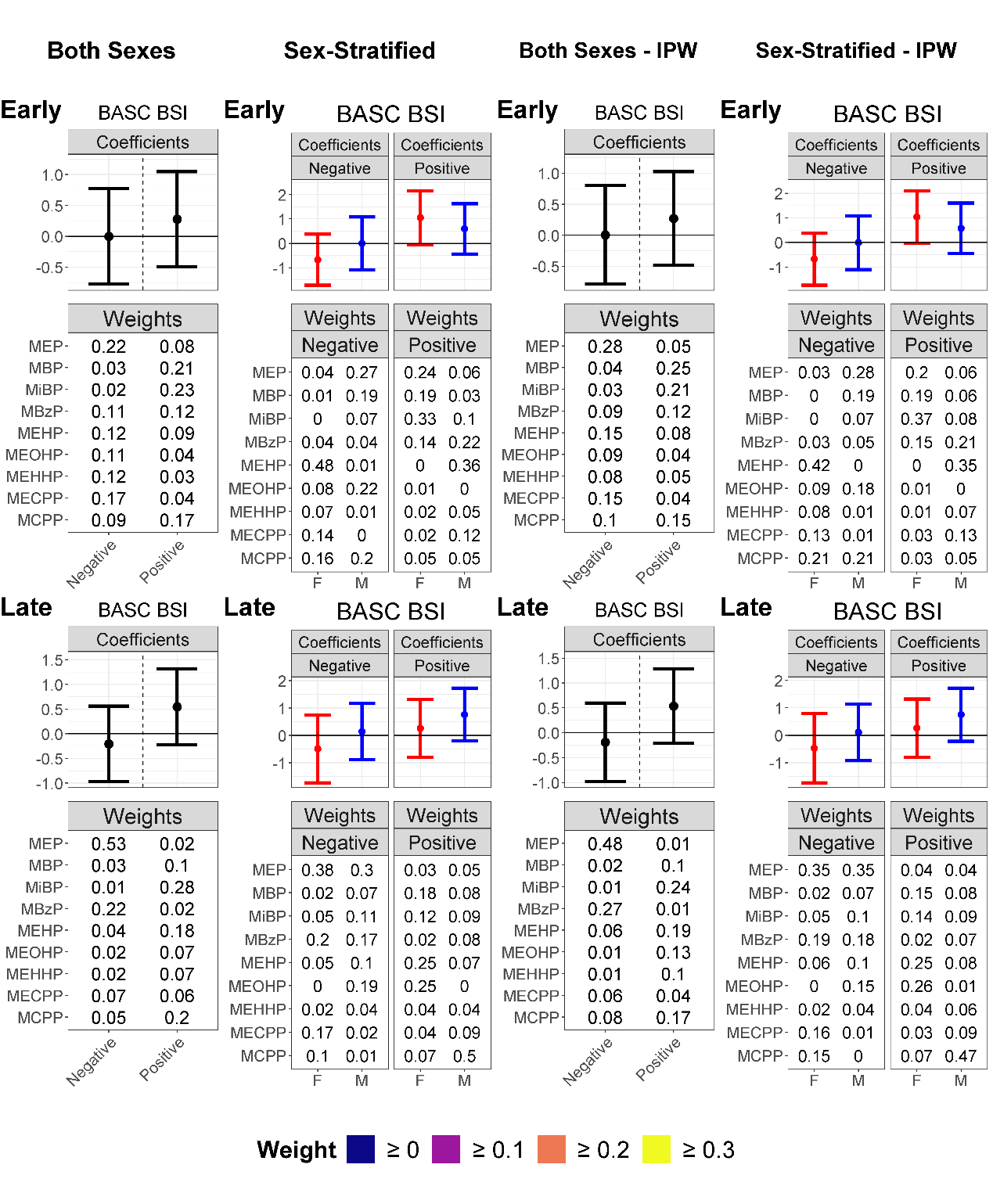


**Results A.6. WQS Regression Coefficients**

The WQS regression coefficients for the weighted quantile sum variable are reproduced in Table A.5 for reference.

Table A.5. WQS Regression Mixture Coefficient Results

| **Model** | **Pregnancy Period** | **Outcome** | **Direction** | **Parameter** | **Coefficient** | **LCI** | **UCI** | **p-value** | **PT p-value** |
| --- | --- | --- | --- | --- | --- | --- | --- | --- | --- |
| Total | Early | BASC-2 | Positive | WQS | 0.41 | -0.32 | 1.15 | 0.27 | 0.51 |
|  |  | EXT | Negative | WQS | 0.05 | -0.71 | 0.81 | 0.90 | 0.87 |
|  |  | BASC-2 | Positive | WQS | -0.13 | -0.93 | 0.68 | 0.76 | 0.93 |
|  |  | INT | Negative | WQS | -0.76 | -1.62 | 0.09 | 0.079 | 0.22 |
|  |  | BASC-2 | Positive | WQS | 0.28 | -0.49 | 1.05 | 0.48 | 0.69 |
|  |  | BSI | Negative | WQS | 0.00 | -0.78 | 0.78 | 1.00 | 0.86 |
|  |  | BASC-2 | Positive | WQS | -0.07 | -0.9 | 0.76 | 0.87 | 0.88 |
|  |  | ADS | Negative | WQS | -0.99 | -1.74 | -0.23 | 0.01 | 0.055 |
|  |  | SRS-2 | Positive | WQS | 0.97 | 0.39 | 1.54 | 0.0009 | 0.01 |
|  |  | TOTAL | Negative | WQS | NA | NA | NA | NA | NA |
|  | Late | BASC-2 | Positive | WQS | 0.66 | -0.07 | 1.39 | 0.075 | 0.19 |
|  |  | EXT | Negative | WQS | NA | NA | NA | NA | NA |
|  |  | BASC-2 | Positive | WQS | 0.57 | -0.19 | 1.34 | 0.14 | 0.39 |
|  |  | INT | Negative | WQS | -0.53 | -1.29 | 0.23 | 0.17 | 0.33 |
|  |  | BASC-2 | Positive | WQS | 0.54 | -0.22 | 1.31 | 0.16 | 0.38 |
|  |  | BSI | Negative | WQS | -0.21 | -0.97 | 0.56 | 0.6 | 0.64 |
|  |  | BASC-2 | Positive | WQS | -0.18 | -0.98 | 0.62 | 0.67 | 0.91 |
|  |  | ADS | Negative | WQS | -0.77 | -1.66 | 0.12 | 0.09 | 0.19 |
|  |  | SRS-2 | Positive | WQS | 0.39 | -0.18 | 0.95 | 0.18 | 0.36 |
|  |  | TOTAL | Negative | WQS | 0.03 | -0.57 | 0.64 | 0.92 | 0.87 |
| Stratified | Early | BASC-2 | Positive | WQS\|M | 0.53 | -0.49 | 1.55 | 0.31 | 0.54 |
|  |  | EXT |  | WQS\|F | 1.30 | 0.33 | 2.28 | 0.0083 | 0.17 |
|  |  |  | Negative | WQS\|M | -0.06 | -1.13 | 1.00 | 0.90 | 0.80 |
|  |  |  |  | WQS\|F | -0.44 | -1.55 | 0.67 | 0.43 | 0.66 |
|  |  | BASC-2 | Positive | WQS\|M | 0.15 | -0.87 | 1.18 | 0.77 | 0.76 |
|  |  | INT |  | WQS\|F | 0.72 | -0.59 | 2.02 | 0.28 | 0.48 |
|  |  |  | Negative | WQS\|M | -0.58 | -1.89 | 0.73 | 0.38 | 0.50 |
|  |  |  |  | WQS\|F | -0.83 | -1.88 | 0.23 | 0.12 | 0.43 |
|  |  | BASC-2 | Positive | WQS\|M | 0.59 | -0.44 | 1.62 | 0.26 | 0.4 |
|  |  | BSI |  | WQS\|F | 1.05 | -0.06 | 2.16 | 0.062 | 0.38 |
|  |  |  | Negative | WQS\|M | -0.01 | -1.1 | 1.08 | 0.99 | 0.78 |
|  |  |  |  | WQS\|F | -0.67 | -1.72 | 0.37 | 0.20 | 0.48 |
|  |  | BASC-2 | Positive | WQS\|M | 0.19 | -0.82 | 1.21 | 0.71 | 0.74 |
|  |  | ADS |  | WQS\|F | 0.12 | -0.96 | 1.19 | 0.83 | 0.83 |
|  |  |  | Negative | WQS\|M | -0.95 | -1.98 | 0.08 | 0.07 | 0.31 |
|  |  |  |  | WQS\|F | -1.54 | -2.61 | -0.47 | 0.0046 | 0.055 |
|  |  | SRS-2 | Positive | WQS\|M | 1.17 | 0.45 | 1.88 | 0.0013 | 0.025 |
|  |  | TOTAL |  | WQS\|F | 1.01 | 0.07 | 1.96 | 0.035 | 0.10 |
|  |  |  | Negative | WQS\|M | NA | NA | NA | NA | NA |
|  |  |  |  | WQS\|F | 0.18 | -0.61 | 0.97 | 0.65 | 0.97 |
|  | Late | BASC-2 | Positive | WQS\|M | 1.32 | 0.40 | 2.25 | 0.0049 | 0.03 |
|  |  | EXT |  | WQS\|F | 0.39 | -0.62 | 1.40 | 0.45 | 0.54 |
|  |  |  | Negative | WQS\|M | NA | NA | NA | NA | NA |
|  |  |  |  | WQS\|F | -0.8 | -1.97 | 0.37 | 0.18 | 0.28 |
|  |  | BASC-2 | Positive | WQS\|M | 0.57 | -0.48 | 1.62 | 0.29 | 0.42 |
|  |  | INT |  | WQS\|F | 0.42 | -0.71 | 1.55 | 0.46 | 0.55 |
|  |  |  | Negative | WQS\|M | -0.44 | -1.53 | 0.64 | 0.42 | 0.58 |
|  |  |  |  | WQS\|F | -0.61 | -1.72 | 0.49 | 0.27 | 0.47 |
|  |  | BASC-2 | Positive | WQS\|M | 0.76 | -0.20 | 1.72 | 0.12 | 0.25 |
|  |  | BSI |  | WQS\|F | 0.26 | -0.80 | 1.32 | 0.63 | 0.72 |
|  |  |  | Negative | WQS\|M | 0.14 | -0.89 | 1.17 | 0.79 | 0.89 |
|  |  |  |  | WQS\|F | -0.49 | -1.73 | 0.75 | 0.43 | 0.47 |
|  |  | BASC-2 | Positive | WQS\|M | 0.14 | -1.02 | 1.30 | 0.82 | 0.76 |
|  |  | ADS |  | WQS\|F | 0.56 | -0.42 | 1.55 | 0.26 | 0.46 |
|  |  |  | Negative | WQS\|M | -0.99 | -2.20 | 0.22 | 0.11 | 0.25 |
|  |  |  |  | WQS\|F | -0.78 | -1.73 | 0.18 | 0.11 | 0.30 |
|  |  | SRS-2 | Positive | WQS\|M | 0.34 | -0.40 | 1.09 | 0.36 | 0.59 |
|  |  | TOTAL |  | WQS\|F | 0.46 | -0.34 | 1.26 | 0.26 | 0.38 |
|  |  |  | Negative | WQS\|M | -0.23 | -1.10 | 0.64 | 0.60 | 0.61 |
|  |  |  |  | WQS\|F | -0.02 | -0.84 | 0.8 | 0.97 | 0.87 |
| IPW - | Early | BASC-2 | Positive | WQS | 0.41 | -0.31 | 1.13 | 0.27 | 0.49 |
| Total |  | EXT | Negative | WQS | 0.04 | -0.72 | 0.81 | 0.91 | 0.87 |
|  |  | BASC-2 | Positive | WQS | -0.15 | -0.96 | 0.65 | 0.71 | 0.92 |
|  |  | INT | Negative | WQS | -0.77 | -1.64 | 0.11 | 0.087 | 0.22 |
|  |  | BASC-2 | Positive | WQS | 0.27 | -0.49 | 1.03 | 0.48 | 0.61 |
|  |  | BSI | Negative | WQS | 0.01 | -0.79 | 0.80 | 0.99 | 0.82 |
|  |  | BASC-2 | Positive | WQS | NA | NA | NA | NA | NA |
|  |  | ADS | Negative | WQS | -0.98 | -1.74 | -0.22 | 0.011 | 0.08 |
|  |  | SRS-2 | Positive | WQS | 0.95 | 0.38 | 1.52 | 0.00099 | 0 |
|  |  | TOTAL | Negative | WQS | NA | NA | NA | NA | NA |
|  | Late | BASC-2 | Positive | WQS | 0.65 | -0.07 | 1.37 | 0.077 | 0.24 |
|  |  | EXT | Negative | WQS | NA | NA | NA | NA | NA |
|  |  | BASC-2 | Positive | WQS | 0.57 | -0.18 | 1.33 | 0.14 | 0.33 |
|  |  | INT | Negative | WQS | -0.53 | -1.29 | 0.23 | 0.17 | 0.38 |
|  |  | BASC-2 | Positive | WQS | 0.53 | -0.21 | 1.28 | 0.16 | 0.33 |
|  |  | BSI | Negative | WQS | -0.19 | -0.98 | 0.60 | 0.63 | 0.64 |
|  |  | BASC-2 | Positive | WQS | -0.19 | -0.99 | 0.62 | 0.65 | 0.95 |
|  |  | ADS | Negative | WQS | -0.77 | -1.65 | 0.12 | 0.089 | 0.13 |
|  |  | SRS-2 | Positive | WQS | 0.39 | -0.15 | 0.93 | 0.16 | 0.34 |
|  |  | TOTAL | Negative | WQS | 0.03 | -0.56 | 0.61 | 0.93 | 0.85 |
| IPW - | Early | BASC-2 | Positive | WQS\|M | 0.51 | -0.53 | 1.55 | 0.33 | 0.42 |
| Stratified |  | EXT |  | WQS\|F | 1.29 | 0.32 | 2.25 | 0.0087 | 0.16 |
|  |  |  | Negative | WQS\|M | -0.06 | -1.13 | 1.02 | 0.91 | 0.8 |
|  |  |  |  | WQS\|F | -0.48 | -1.60 | 0.64 | 0.40 | 0.62 |
|  |  | BASC-2 | Positive | WQS\|M | 0.14 | -0.89 | 1.17 | 0.79 | 0.74 |
|  |  | INT |  | WQS\|F | 0.72 | -0.55 | 1.99 | 0.26 | 0.54 |
|  |  |  | Negative | WQS\|M | -0.59 | -1.92 | 0.74 | 0.38 | 0.49 |
|  |  |  |  | WQS\|F | -0.8 | -1.87 | 0.26 | 0.14 | 0.46 |
|  |  | BASC-2 | Positive | WQS\|M | 0.58 | -0.45 | 1.61 | 0.27 | 0.48 |
|  |  | BSI |  | WQS\|F | 1.03 | -0.04 | 2.10 | 0.057 | 0.27 |
|  |  |  | Negative | WQS\|M | -0.01 | -1.1 | 1.08 | 0.99 | 0.83 |
|  |  |  |  | WQS\|F | -0.68 | -1.74 | 0.39 | 0.21 | 0.53 |
|  |  | BASC-2 | Positive | WQS\|M | 0.14 | -0.88 | 1.16 | 0.78 | 0.82 |
|  |  | ADS |  | WQS\|F | 0.05 | -1.07 | 1.17 | 0.93 | 0.88 |
|  |  |  | Negative | WQS\|M | -0.93 | -1.97 | 0.11 | 0.077 | 0.18 |
|  |  |  |  | WQS\|F | -1.51 | -2.57 | -0.45 | 0.0048 | 0.065 |
|  |  | SRS-2 | Positive | WQS\|M | 1.16 | 0.44 | 1.88 | 0.0014 | 0.025 |
|  |  | TOTAL |  | WQS\|F | 1 | 0.05 | 1.95 | 0.039 | 0.11 |
|  |  |  | Negative | WQS\|M | NA | NA | NA | NA | NA |
|  |  |  |  | WQS\|F | 0.19 | -0.60 | 0.98 | 0.64 | 0.97 |
|  | Late | BASC-2 | Positive | WQS\|M | 1.29 | 0.36 | 2.23 | 0.0061 | 0.046 |
|  |  | EXT |  | WQS\|F | 0.39 | -0.61 | 1.4 | 0.44 | 0.62 |
|  |  |  | Negative | WQS\|M | NA | NA | NA | NA | NA |
|  |  |  |  | WQS\|F | -0.79 | -2.00 | 0.41 | 0.19 | 0.35 |
|  |  | BASC-2 | Positive | WQS\|M | 0.57 | -0.47 | 1.61 | 0.28 | 0.46 |
|  |  | INT |  | WQS\|F | 0.42 | -0.71 | 1.55 | 0.46 | 0.53 |
|  |  |  | Negative | WQS\|M | -0.47 | -1.57 | 0.63 | 0.40 | 0.54 |
|  |  |  |  | WQS\|F | -0.57 | -1.68 | 0.54 | 0.31 | 0.50 |
|  |  | BASC-2 | Positive | WQS\|M | 0.75 | -0.22 | 1.71 | 0.13 | 0.26 |
|  |  | BSI |  | WQS\|F | 0.26 | -0.80 | 1.33 | 0.63 | 0.73 |
|  |  |  | Negative | WQS\|M | 0.11 | -0.92 | 1.13 | 0.84 | 0.91 |
|  |  |  |  | WQS\|F | -0.47 | -1.73 | 0.79 | 0.46 | 0.46 |
|  |  | BASC-2 | Positive | WQS\|M | 0.17 | -0.98 | 1.32 | 0.77 | 0.72 |
|  |  | ADS |  | WQS\|F | 0.56 | -0.42 | 1.54 | 0.26 | 0.43 |
|  |  |  | Negative | WQS\|M | -0.99 | -2.19 | 0.22 | 0.11 | 0.26 |
|  |  |  |  | WQS\|F | -0.79 | -1.76 | 0.19 | 0.11 | 0.27 |
|  |  | SRS-2 | Positive | WQS\|M | 0.34 | -0.41 | 1.09 | 0.37 | 0.57 |
|  |  | TOTAL |  | WQS\|F | 0.46 | -0.33 | 1.26 | 0.25 | 0.42 |
|  |  |  | Negative | WQS\|M | -0.25 | -1.1 | 0.60 | 0.56 | 0.66 |
|  |  |  |  | WQS\|F | -0.01 | -0.84 | 0.81 | 0.97 | 0.78 |

For the column “Model”, Total = WQS regressions run on the total population, Stratified = WQS regressions stratified by child sex, and IPW = WQS regressions with inverse probability weighting. PT p-value = permutation test p-value, WQS|M = male-specific stratified WQS coefficients, WQS|F = female-specific stratified WQS coefficients, LCI = lower 95% confidence interval, UCI = upper 95% confidence interval, EXT = externalizing, INT = internalizing, BSI = behavioral symptoms index, and ADS = adaptive skills.

**Results A.7. Individual Phthalate Linear Quintile Regression Coefficients**

This section includes the results for linear regressions in which each outcome is regressed on each individual phthalate metabolite concentration transformed into study center-specific quintiles and with the same set of covariates used in the WQS regressions. The results for the total study population are shown in Figure A.10. Sex-stratified results for these individual phthalate regressions can be found in Figure A.11 for females and Figure A.12 for males. All regression coefficients represent the unit change in outcome T score per 1 quintile increase in each individual phthalate. Significant coefficients are marked with a star above the upper confidence interval. Most of the individual regressions are not significant, but the coefficient means for most phthalates trend in the same direction for those mixtures with significant coefficients in the WQS regression models. These results highlight the importance of evaluating the cumulative associations of phthalate mixtures rather than each component of the mixture individually.

Figure A.10. All Individual Phthalate Regression Coefficient Means and 95% CIs in either Early or Late Pregnancy for the Total Study Population


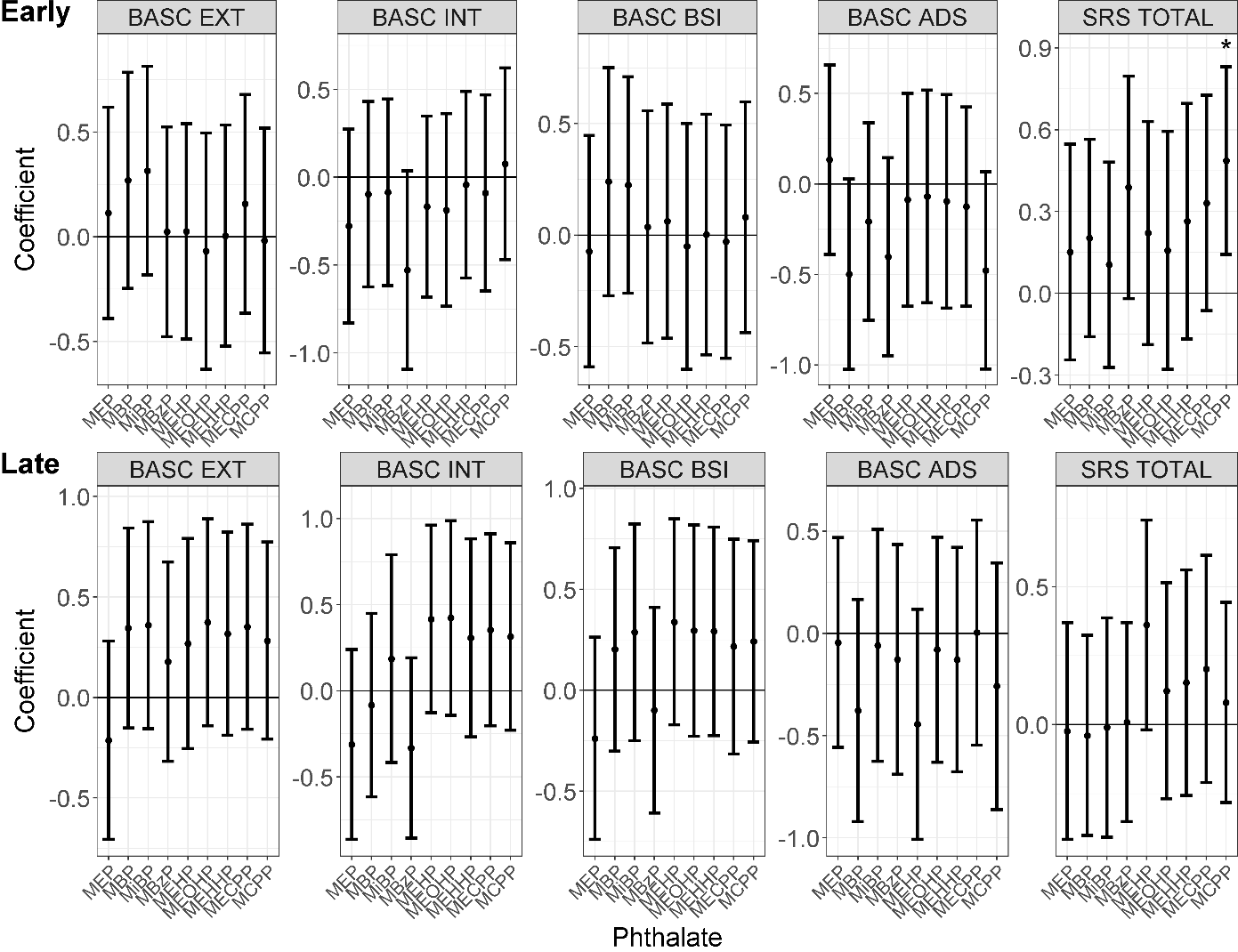


Figure A.11. All Female-Specific Sex-Stratified Individual Phthalate Regression Coefficient Means and 95% CIs in either Early or Late Pregnancy


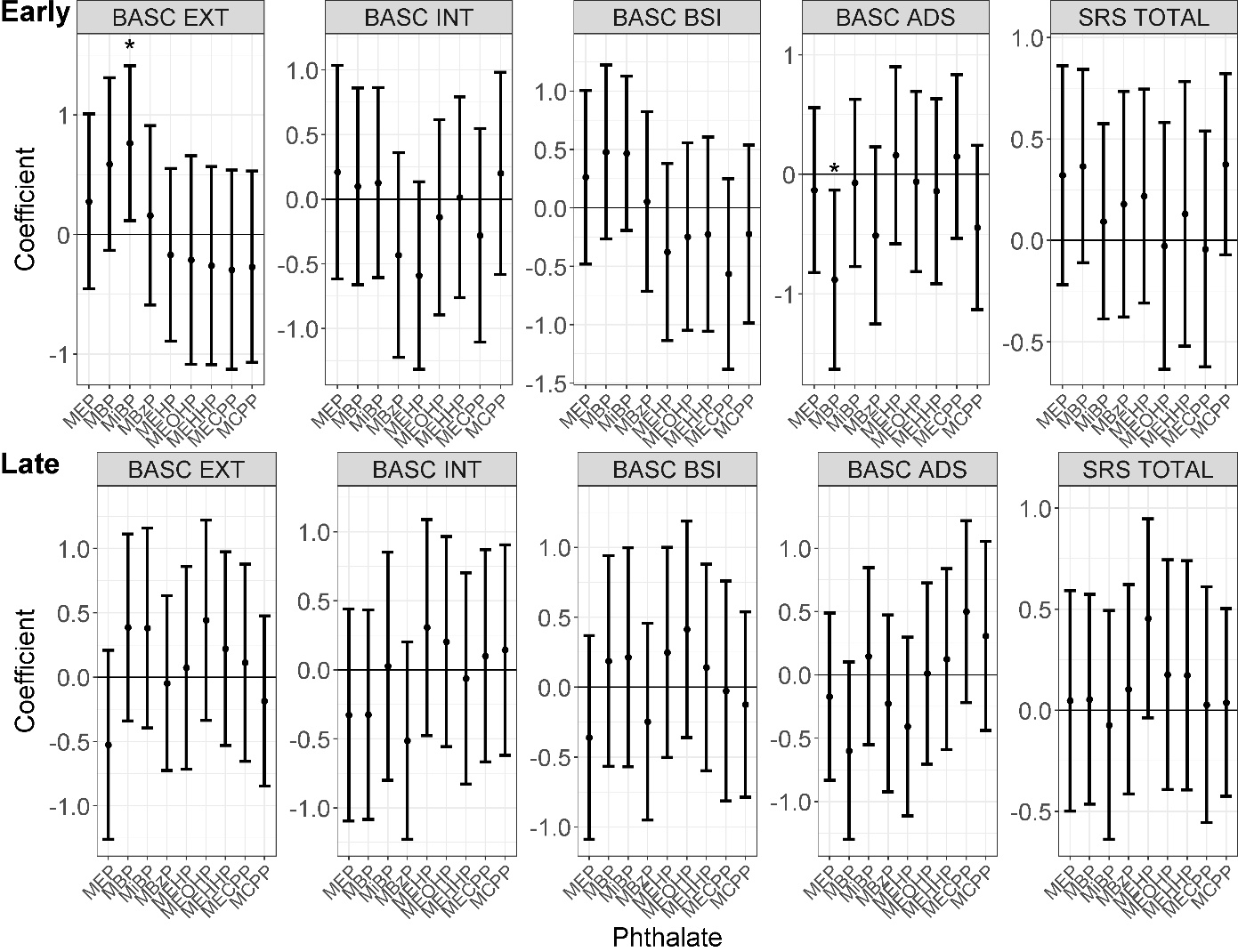


Figure A.12. All Male-Specific Sex-Stratified Individual Phthalate Regression Coefficient Means and 95% CIs in either Early or Late Pregnancy


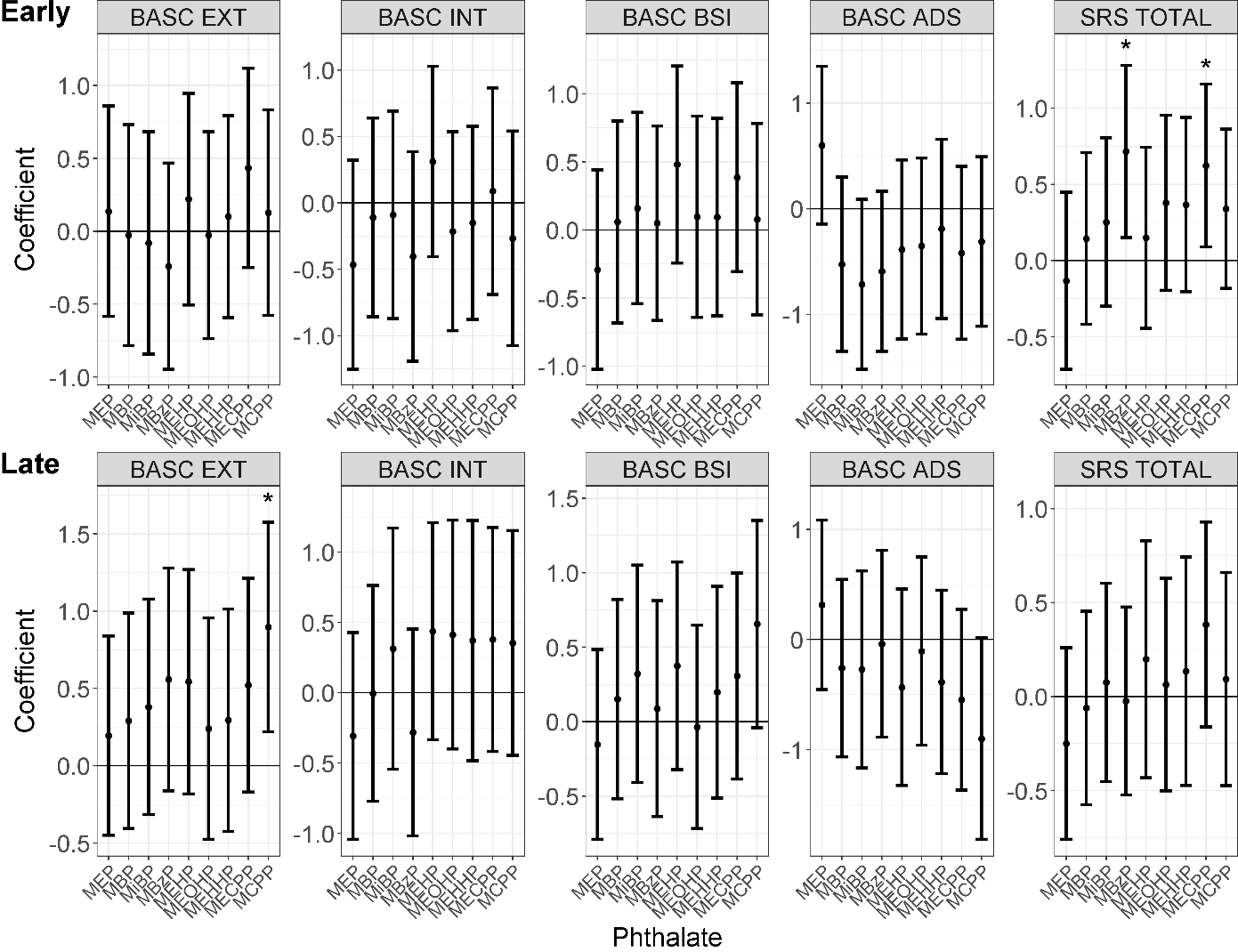


**References**

Seaman SR, White IR. 2013. Review of inverse probability weighting for dealing with missing data. Stat Methods Med Res 22:278-295.
